# Supplementary material for: Patterns and correlates of active commuting in adults with type 2 diabetes: cross-sectional evidence from UK Biobank
Source: BMJ Open. 2017 Oct 8;7(10):e017132. doi: 10.1136/bmjopen-2017-017132 (PMC5640023; doi:10.1136/bmjopen-2017-017132)
Supplement: Author's manuscript [file bmjopen-2017-017132.draft_revisions.pdf]

BMJ Open is committed to open peer review. As part of this commitment we make the peer review history of every article we publish publicly available.

When an article is published we post the peer reviewers' comments and the authors' responses online. We also post the versions of the paper that were used during peer review. These are the versions that the peer review comments apply to.

The versions of the paper that follow are the versions that were submitted during the peer review process. They are not the versions of record or the final published versions. They should not be cited or distributed as the published version of this manuscript.

BMJ Open is an open access journal and the full, final, typeset and author-corrected version of record of the manuscript is available on our site with no access controls, subscription charges or pay-per-view fees (http://bmjopen.bmj.com).

If you have any questions on BMJ Open's open peer review process please email editorial.bmjopen@bmj.com

BMJ Open

Patterns and correlates of active commuting in adults with type 2 diabetes: cross-sectional evidence from UK Biobank

Journal:	*BMJ Open*
Manuscript ID	bmjopen-2017-017132
Article Type:	Research
Date Submitted by the Author:	03-Apr-2017
Complete List of Authors:	Falconer, Catherine; Gloucestershire Hospitals NHS Foundation Trust, ; University of Bristol, NIHR Bristol Nutrition Biomedical Research Unit<br>Cooper, Ashley; University of Bristol<br>Flint, Ellen; London School of Hygiene and Tropical Medicine, Social and Environmental Health Research
**Primary Subject Heading**:	Public health
Secondary Subject Heading:	Sports and exercise medicine
Keywords:	General diabetes < DIABETES & ENDOCRINOLOGY, EPIDEMIOLOGY, PREVENTIVE MEDICINE, PUBLIC HEALTH

SCHOLARONE™
Manuscripts

Title Page

Patterns and correlates of active commuting in adults with type 2 diabetes: cross-sectional evidence from UK Biobank

Falconer, Catherine L[1,2]; Cooper, Ashley R[2,3]; Flint, Ellen[4]

[1]NHS Gloucestershire Foundation Trust,

[2]NIHR Bristol Nutrition Biomedical Research Unit, University of Bristol

[3]Centre for Exercise, Nutrition and Health Sciences, University of Bristol

[4] Department of Social and Environmental Health Research, London School of Hygiene and Tropical Medicine

For peer review only

Word Count: 2,920
Abstract: 250
Number of tables: 3
Number of figures: 0

Corresponding author:

Professor Ashley Cooper
Bristol Nutrition BRU
Level 3, University Hospitals Research and Education Centre,
Upper Maudlin Street,
Bristol,
BS2 8AE
Email: Ashley.cooper@bristol.ac.uk

ABSTRACT

Objectives To describe the active commuting patterns of adults with type 2 diabetes and how these relate to physical activity and sedentary behaviour. Social and environmental correlates of active commuting will also be explored.

Design Cross-sectional analysis of a cohort study

Settings Population cohort of over 500,000 people recruited from 22 centres across the United Kingdom. Participants aged between 37 and 73 years were recruited between 2006 and 2010.

Participant 6,896 participants with a self-reported diabetes diagnosis who reported commuting to work and had complete covariate data were included in the analysis.

Outcome measures Outcome measures were weekly minutes of moderate-to-vigorous physical activity (MVPA), and hours/day of sedentary time, and participation in active travel.

Results AC was reported by 5.5% of participants with the overwhelming majority using the car to commute (80%). AC was associated with an additional 73 (95%CI 10.8, 134.9) and 105 (95%CI 41.7, 167.2) weekly minutes of MVPA for males and females respectively. AC was associated with reduced sedentary time (β-1.1 95%CI -1.6, -0.7 hours/day for males and β-0.8 95%CI -1.2, -0.3 hours/day for females). Deprivation and distance from home to work were identified as correlates of AC behaviour.

Conclusions Rates of active commuting are very low in adults with type 2 diabetes. However, active commuting offers a potentially sustainable solution to increasing physical activity and reducing sedentary behaviour. Therefore, strategies to improve the environment and encourage active commutinig may help to increase population levels of physical activity and reduce the disease burden associated with type 2 diabetes.

For peer review only

Article summary

Strengths and limitations of this study

This study uses the UK Biobank to examine the active commuting behaviours of adults with type 2 diabetes. UK Biobank is a large, geographically diverse cohort which provides the largest exploration of active commuting in this population to date.

UK Biobank provided the opportunity to be able to explore a multitude of employment, social and environmental factors which may contribute to active commuting.

We were unable to distinguish between walking and cycling behaviour, and also were unable to explore different type of public transport modes for example bus or train.

The analysis used in this study is cross-sectional and therefore we cannot infer causality.

This study is further limited by the use of self-report measures of physical activity and sedentary time.

For peer review only

For peer review only - http://bmjopen.bmj.com/site/about/guidelines.xhtml

INTRODUCTION

Type 2 diabetes mellitus (T2DM) is a disease primarily driven by unhealthy lifestyle behaviours, such as poor diet and physical inactivity [1] and displays a social gradient [2]. The worldwide age-standardised prevalence of T2DM has doubled to 8.5% since 1980 [3]. In the UK the prevalence is 6% and the ongoing treatment and management burden of T2DM and its associated complications places a considerable burden on the National Health Service [4,5].

The role of physical activity (PA) in the prevention and management of T2DM is well understood [6]. However, few adults with T2DM achieve UK Government recommendations of at least 150 minutes moderate-to-vigorous physical activity (MVPA) per week [7] and many interventions fail to achieve increases in PA sufficient to confer metabolic benefits [7–9]. Sedentary behaviour is also an independent risk factor for the development of T2DM [10]. Cross-sectional evidence from UK Biobank suggests that adults with T2DM are characterised by a phenotype of unhealthy behaviours including prolonged sedentary time, physical inactivity and poor sleep, suggesting that these behaviours should be targeted in parallel [11].

The role of active travel in increasing PA and controlling excessive weight gain has been recognised and endorsed by the National Institute for Health and Care Excellence (NICE) [12]. Active travel has the potential to increase individuals PA and reduce sedentary time, and more widely to lower levels of congestion and noise and air pollution [13], and can be sustainably incorporated into daily living [14]. However, rates of AC are low in the UK; of the 58% of adults who commute to work, 67% use private motorised transport and 17.8% use public transport, with only 10.9% walking and 3.1% commuting by bicycle [15].

There is increasing cross-sectional evidence of an association between AC and measures of obesity [16,17]. The UK Understanding Society study found that commuting by walking, cycling or public transport was predictive of lower BMI and body fat percentage [16]. In a separate analysis of the same dataset, the adjusted odds of reporting a diabetes diagnosis were 0.60 (95% CI 0.43, 0.83) in those who walked to work and 0.50 (95% CI 0.27, 0.93) in those who cycled, compared to car users [18]. Similarly in the UK Biobank study, AC was significantly and independently associated with lower BMI and body fat percentage [17]. The protective effect of AC is likely to be mediated through increased PA as demonstrated in a UK study which used accelerometry and GPS receivers to show that walking to work contributed 47.3% of daily MVPA [19].

Causal evidence from longitudinal or experimental studies is rare. A longitudinal analysis of the UK Biobank cohort looked at changes in BMI in response to changes in commuting

For peer review only - http://bmjopen.bmj.com/site/about/guidelines.xhtml

mode over a 4 year follow up. Individuals who transitioned from car commuting at baseline to active or public transportation modes at follow up had a decrease in BMI of -0.30kg/m^2 (95%CI -0.47, -0.13) [20]. Similarly, a prospective study exploring the influence of cycling demonstrated a lower BMI in those who maintained cycling over a 12 month follow up compared to those who never cycled to work [21].

Promoting AC may help to increase population levels of activity; however, there is a need to understand the correlates of AC to develop appropriate interventions. Some key determinants of commuter cycling and walking behaviour have been identified as high population density, dedicated cycling paths, short trip distances and good traffic and road conditions [22][23]. Most work to date has been performed in healthy adults and there is a need to understand how these findings relate to populations with T2DM who are characterised by obesity and physical inactivity.

The primary aim of this work is to describe the AC patterns of adults with T2DM in UK Biobank. Specifically, the study explores how AC is associated with PA and sedentary time in this population (objective 1). The study will also aim to identify social and environmental factors (age, social class and traffic density) that may predict participation in AC (objective 2).

METHODS

Study population

Baseline data from the UK Biobank study were used (project 19307). UK Biobank is a large population study which aims to examine how the environment, lifestyle and genes interact to affect health [24]. Full recruitment procedures are described in detail elsewhere, [25] and briefly summarised below.

Between 2006 and 2010, recruitment invitations were sent to 9.2million people from 22 assessment centres around the UK to obtain a sample of 502,565 adults aged 37-73 years [11]. Following recruitment, participants provided written, informed consent and attended an assessment centre for data collection. A touchscreen questionnaire covered socio-demographics, occupation, lifestyle, early exposures, cognitive function, family history and medical history. Participants also underwent a verbal interview, physical measures and provided a blood/urine sample. All data were anonymised. The UK Biobank Access Subcommittee granted approval for the data access under a Material Transfer Agreement with the London School of Hygiene and Tropical Medicine who provided ethical approval for this project (LSHTM Ethics reference: 10838).

For peer review only - http://bmjopen.bmj.com/site/about/guidelines.xhtml

Study sample

Participants who reported having diabetes or T2DM were selected (n=26,408). We excluded anyone <35 years at diagnosis of diabetes and who reported taking insulin within the first year of diagnosis to reduce the likelihood of type 1 or monogenic forms of diabetes [11], (n=20,143). Only participants who reported commuting behaviours and had complete covariate data were included in the analysis (n=6,896).

Active commuting measures

In response to the question "what types of transport do you use to get to and from work?", participants could select one or more of the following options: car/motor vehicle; walk; public transport; cycle. For this study, two AC exposure variables were derived: 1. a three-category exposure variable; (1) car or car and public transport; (2) public transport and mixed; (3) walking or cycling exclusively and 2. a binary variable for AC ((1) Not active commuting; (2) Active commuting (walking or cycling only)).

Physical activity

PA was assessed using questions adapted from the validated short form of the International Physical Activity Questionnaire (IPAQ) [26]. The questions asked participants to self-report the frequency, duration and intensity of walking, moderate and vigorous activity. Total physical activity was derived by weighting time spent in vigorous, moderate and walking activity by the relevant energy expended for each activity to produce metabolic equivalents (MET) minutes/week of PA [27]. MVPA was derived as the sum of reported duration and frequency of moderate and vigorous activity. From this, a variable for 'attainment of physical activity guidelines' (150 minutes' moderate PA or 75 minutes of vigorous activity per week) was derived[7].

Sedentary behaviour

Self-reported television viewing, computer use and driving behaviour were collected from the touchscreen questionnaire. An estimate of average total sedentary time was derived by summing the daily reported duration of television viewing, computer use and driving. Total sedentary time is expressed as hours per day.

Environmental correlates

Local environmental exposure variables were collated by the Small Area Health Statistics Unit [28] as part of the BioSHaRE-EU Environmental Determinants of Health Project [29]. Air pollution estimates were modelled for the year 2010 using a Land Use Regression (LUR) model developed as part of the European Study of Cohorts for Air Pollution Effects (ESCAPE) [30]. ESCAPE estimates of particulate matter (PM) are valid up to 400km from

For peer review only - http://bmjopen.bmj.com/site/about/guidelines.xhtml

the monitoring area, which in this case was Greater London. Therefore, participants who have an address more than 400km from this site were not assigned a value for PM2.5-10. Traffic variables, including inverse distance to a major road and traffic density, were also calculated using the LUR model, based on Eurostreets (v3.1) digital road network for the year 2008.

Covariates

A range of confounding variables were considered. Socioeconomic covariates were; Townsend Deprivation Index of residential area, annual gross household income, highest educational qualification. Further demographic covariates were: age (years); sex; ethnicity, census-derived residential area population density classification (urban/fringe, rural). The potential confounding effects of occupational physical activity were considered: job involves standing/walking; job involves heavy manual/physical work, shift work, non-work related transport modes (active modes: yes/no). Health covariates were: poor self-rated health (yes/no). Commute frequency (number of outward journeys per week, truncated at 10) and distance between home and workplace (reported in miles) were included as covariates in the model.

Statistical analyses

Descriptive analyses were undertaken to describe the commuting patterns of the population, the prevalence of PA and sedentary behaviours and the patterning of hypothesised confounding variables.

To address objective 1, multivariate linear regression was used to describe the association between AC and each of the two outcome variables (PA and sedentary time) adjusting for potential confounding variables in two step-wise series of three nested models. The first model presents the unadjusted association between the exposure (commuting mode) and outcome of interest. The second model adjusts for demographic and socio-economic factors. The third model additionally adjusts for employment-related confounders such as distance from home to work.

To address objective 2, multivariate logistic regression modelling was used to explore potential socio-demographic, environmental and employment-related correlates of AC behaviour. The model used was adjusted for all included variables. All analyses controlled for geographical clustering by assessment centre, and potential interactions by gender, ethnicity and household income were explored. For these analyses, the outcome was expressed as a binary categorical variable ((1) not active commuting, (2) active commuting). All analyses were conducted using Stata v14 [31].

For peer review only - http://bmjopen.bmj.com/site/about/guidelines.xhtml

RESULTS

The final sample was 6,896 participants with T2DM who reported commuting behaviour and provided complete data on all covariates (Table I).

Table 1. Demographic, commuting behaviour, employment, physical activity and environmental characteristics of the study population

Variable	Males (n=4,473)	Females (n=2,423)	Total (n=6,896)
**Demographics**			
Age (years)	56.7 ± 6.5	54.53 ± 6.6	55.9 ± 6.6
Ethnicity			
White British	81.8	78.2	80.5
Other white	5.3	6.5	5.7
South Asian	6.2	4.2	5.5
Black Caribbean	1.3	3.9	2.2
Black African	1.8	2.1	1.9
Chinese	0.3	0.6	0.4
Mixed ethnicity	0.6	0.8	0.7
Other ethnicity	2.7	3.8	3.1
Townsend Index of deprivation(quintiles)			
1	16.1	14.1	15.4
2	17.2	15.8	16.7
3	19.6	20.9	20.1
4	21.2	22.1	21.8
5	25.5	27.1	26.0
Highest Educational Qualification			
University	29.6	30.8	30.0
A Levels/AS Levels	11.2	11.2	11.4
GCSE/O Levels	20.5	25.0	22.1
CSE's or equivalent	6.4	7.9	6.9
NVQ or HND or HNC	11.5	7.0	9.9
Other professional qualifications	5.32	6.2	5.6
None	15.6	11.6	14.2
Average household income			
<£18,000	12.2	20.5	15.1
£18,000 to £30,999	27.4	28.0	27.6
£31,000 to £51,999	30.9	29.3	30.4
£52,000 to £100,000	23.8	18.6	22.0
>£100,000	5.7	3.5	4.9
Poor self-rated health	9.9	9.0	9.6
**Employment**			
Job involves heavy lifting			
Never/rarely	59.8	65.8	61.9
Sometimes	25.0	22.5	24.1
Usually	8.1	5.7	7.2
Always	7.1	6.0	6.7
Job involves walking			
Never/rarely	31.3	32.8	31.8
Sometimes	32.7	31.2	32.2
Usually	16.1	13.8	15.3
Always	19.9	22.2	20.7
Shift work	7.7	5.9	7.1
**Commuting behaviour**			
Commute frequency (no. outward journeys)	4.6 ± 2.1	4.3 ± 1.8	4.5 ± 2.0
Distance from home to work (miles)	14.9 ± 28.4	7.5 ± 9.9	12.3 ± 24.0
Commuting mode			
Car and car and public transport	83.2	75.3	80.4
Mixed or public transport	12.3	17.5	14.1

For peer review only - http://bmjopen.bmj.com/site/about/guidelines.xhtml

Walking or cycling only	4.5	7.2	5.4
Non work active travel	40.4	40.2	40.3
**Physical activity and sedentary time**			
Daily sedentary time (hours)	6.0 ± 3.1	4.9 ± 2.7	5.6 ± 3.0
Total weekly MVPA[1] (minutes)	263.9 ± 446.4	214.9 ± 383.4	246.6 ± 425.9
Total weekly MET[2]-minutes	2373.3 ± 3099.8	2014.7 ± 2854.7	2245.5 ± 3106.2
Achieves PA guidelines[3]	54.3	53.8	54.1
**Environmental characteristics**			
Close to major road	8.0	8.2	8.1
Population density (urban/fringe)	94.5	95.7	94.9
Inverse distance to nearest road (1/m)	0.05 (0.05, 0.05)	0.05 (0.05, 0.05)	0.05 (0.05, 0.05)
Air pollution PM2.5-10 (micro-g/m^3)	6.5 ± 0.9	6.5 ± 0.9	6.5 ± 0.9
Traffic density (vehicles/day)	1699.4 ± 5663.6	1716.2 ± 6057.0	1698.6 ± 5663.6

[1]MVPA, Moderate to vigorous physical activity; [2]MET, metabolic equivalents (1MET=1kcal/kg/hr); [3]PA guidelines, physical activity guidelines (150 minutes MVPA/week or 75 minutes vigorous activity/week)[7]

Car travel was reported by 83.2% of male and 75.2% of female participants, with walking or cycling exclusively reported by 4.5% of males and 7.2% of females. Females spent less time sedentary than males (4.9 ± 2.7 hours/day versus 6.0 ± 3.1 hours/day), while males reported more weekly MVPA (263.9 ± 446.4 minutes/week versus 214.9 ± 383.4 minutes/week).

The results of analyses to investigate the association between commuting mode, PA and sedentary behaviour (objective 1) are displayed in table II. A significant interaction for gender was found so stratified analyses are presented.

For peer review only

Table 2. Results of linear regression modelling to investigate the associations between commuting behaviour, physical activity and sedentary time, stratified by gender.

	MVPA[1]		Total MET minutes		Total sedentary time (hours)	
	**Males β (95% CI)**	**Females β (95% CI)**	**Males β (95% CI)**	**Females β (95% CI)**	**Males β (95% CI)**	**Females β (95% CI)**
**Model 1**						
Car (reference)	0.0	0.0	0.0	0.0	0.0	0.0
Mixed/PT[2]	-49.9 (-89.8, -10.1)	12.5 (-27.9, 52.8)	93.0 (-381.0, 193.0)	312.3 (12.3, 612.4)	-2.0 (-2.3, -1.7)	-1.0 (-1.3, 0.7)
Active travel	86.4 (23.1, 149.7)	156.2 (97.0, 215.4)	777.0 (320.6, 1233.3)	1287.3 (847.3, 1727.4)	-1.3 (-1.8, -0.9)	-1.0 (-1.4, -0.6)
**Model 2[3]**						
Car (reference)	0.0	0.0	0.0	0.0	0.0	0.0
Mixed/PT	-57.4 (-98.6, -16.2)	-3.0 (-45.9, 40.1)	-156.1 (-452.4, 140.3)	155.2 (-163.4, 473.9)	-2.2 (-2.4, -1.9)	-1.2 (-1.5, -0.9)
Active travel	74.6 (10.9, 138.4)	145.3 (85.5, 205.2)	665.5 (207.0, 1124.1)	1149.4 (706.3, 1592.5)	-1.5 (-1.9, -1.0)	-1.1 (-1.5, -0.7)
**Model 3[4]**						
Car (reference)	0.0	0.0	0.0	0.0	0.0	0.0
Mixed/PT	-29.6 (-70.6, 11.4)	11.5 (-35.3, 58.3)	47.0 (-240.9, 334.9)	290.8 (-46.0, 627.6)	-2.2 (-2.5, -1.9)	-1.1 (-1.5, -0.8)
Active travel	72.9 (10.8, 134.9)	104.5 (41.7, 167.2)	586.6 (150.9, 1022.3)	722.3 (270.3, 1174.1)	-1.1 (-1.6, -0.7)	-0.8 (-1.2, -0.3)

[1]MVPA: Moderate to vigorous physical activity; [2]Mixed/PT: mixed modes of transport and public transport; [3]Model is adjusted for age, ethnicity, average household income, Townsend deprivation score, assessment centre, poor health; [4]additionally adjusted for distance from home to work, commute frequency, population density, non-work active travel and job involves walking.

For peer review only - http://bmjopen.bmj.com/site/about/guidelines.xhtml

In all models, AC was associated with higher MVPA. In fully adjusted models, compared to car use, AC was associated with an additional 72.9 (95%CI 10.8, 134.9) minutes/week of MVPA for males and 104.5 (95%CI 41.7, 167.2) minutes/week for females. The association with mixed and public transport was less clear. In males, there was a suggestion that mixed and public transport modes were associated with less MVPA compared to car use. This association was attenuated once environmental factors were included in the model (adjusted β 29.6; 95%CI -70.6, 11.4).

AC was also consistently associated with increased total PA in both genders, an effect which survived adjustment for all confounding factors (β 586.6; 95%CI 150.9, 1022.3 for males and β 722.3; 95%CI 270.3, 1174.1 for females). In unadjusted models, mixed and public transport modes were associated with increased total PA for females only; however, this was attenuated once employment and demographic factors were included in the model.

In both males and females, and in all models, consistent associations were seen between commuting mode and total sedentary time. In fully adjusted models, compared to male car users, AC was associated with 1.1 (95%CI -1.6, -0.7) fewer hours/day sedentary while mixed and public transport modes were associated with 2.2 (95%CI -2.5, -1.9) fewer sedentary hours/day. A similar effect was seen in females with AC associated with 0.8 (95%CI -1.2, -0.3) fewer sedentary hours/day and mixed and public transport modes were associated with 1.1 (95%CI -1.5, -0.8) fewer sedentary hours/day compared to car users.

The results of multivariate logistic regression performed to identify social, employment and environmental correlates of AC behaviour (objective 2) are displayed in Table III. There was evidence of a significant interaction for gender so stratified results are presented.

Table 3. Results of logistic regression modelling to identify potential social, employment and environmental correlates of active travel.

	Model 1 Individual factors		Model 2 Employment factors		Model 3 Environmental factors	
	**Males OR (95% CI)**	**Females OR (95% CI)**	**Males OR (95% CI)**	**Females OR (95% CI)**	**Males OR (95% CI)**	**Females OR (95% CI)**
**Social factors**						
Age (years)	1.0 (0.9, 1.0)	0.98 (0.95, 1.00)	0.98 (0.96, 1.00)	0.97 (0.94, 0.99)	1.01 (0.98, 1.03)	0.97 (0.95, 0.99)
Townsend Deprivation quintile						
1 (ref)	1.0	1.0	1.0	1.0	1.0	1.0
2	1.13 (0.58, 2.22)	2.09 (0.93, 4.71)	0.88 (0.42, 1.84)	2.76 (1.10, 6.92)	1.19 (0.60, 2.37)	1.99 (0.88, 4.50)
3	1.90 (1.03, 3.49)	3.00 (1.42, 6.37)	1.35 (0.69, 2.64)	2.54 (1.10, 5.90)	1.80 (0.96, 3.37)	2.69 (1.26, 5.75)
4	2.04 (1.12, 3.71)	3.25 (1.55, 6.83)	1.49 (0.77, 2.88)	2.60 (1.14, 5.95)	1.79 (0.96, 3.34)	2.92 (1.38, 6.19)
5	4.24 (2.41, 7.44)	3.34 (1.61, 6.94)	2.28 (1.23, 4.21)	3.07 (1.36, 6.94)	3.32 (1.84, 6.01)	3.08 (1.47, 6.48)
Average household						

For peer review only - http://bmjopen.bmj.com/site/about/guidelines.xhtml

income (£) *<£18,000 (ref)*	1.0	1.0	1.0	1.0	1.0	1.0
£18,000 to £30,999 *£31,000 to £51,999*	0.68 (0.44, 1.04)	0.72 (0.48, 1.09)	0.97 (0.59, 1.59)	1.25 (0.77, 2.04)	0.61 (0.39, 0.97)	0.76 (0.50, 1.16)
£52,000 to £100,000	0.69 (0.44, 1.07)	0.39 (0.24, 0.62)	1.23 (0.74, 2.04)	0.94 (0.53, 1.65)	0.65 (0.42, 1.03)	0.41 (0.25, 0.67)
>£100,000	0.85 (0.53, 1.37)	0.35 (0.20, 0.62)	1.34 (0.77, 2.33)	0.88 (0.45, 1.74)	0.80 (0.49, 1.32)	0.37 (0.21, 0.67)
	1.10 (0.58, 2.11)	0.90 (0.40, 2.01)	1.27 (0.59, 2.79)	4.27 (1.47, 12.40)	0.90 (0.43, 1.89)	0.75 (0.30, 1.87)
Assessment centre	0.97 (0.95, 1.00)	1.00 (0.98, 1.03)	0.98 (0.95, 1.01)	1.02 (0.98, 1.05)	0.98 (0.95, 1.00)	1.00 (0.97, 1.03)
Poor health	0.70 (0.41, 1.18)	0.83 (0.47, 1.46)	0.56 (0.31, 1.00)	1.04 (0.53, 2.04)	0.62 (0.35, 1.11)	0.89 (0.50, 1.56)
**Employment characteristics**						
Distance from home to work (miles)				1.0		
<1.5 (ref)			1.0	0.07 (0.05, 0.11)		
			0.12 (0.08, 0.17)			
1.5-4				0.01 (0.05, 0.03)		
			0.01 (0.01, 0.03)			
4-10				0.00		
>10			0.002 (0.001, 0.01)			
Commute frequency			0.95 (0.86, 1.05)	1.01 (0.91, 1.14)		
Job involves walking			0.97 (0.83, 1.13)	1.20 (1.00, 1.43)		
Shift work			0.85 (0.43, 1.67)	0.76 (0.31, 1.88)		
**Environmental characteristics**						
Population density (ref: urban)					0.12 (0.02, 0.90)	0.39 (0.09, 1.62)
Air pollution (PM10 microg/m3)					1.01 (0.84, 1.21)	0.89 (0.72, 1.09)
Traffic density (no. cars)					1.0 (0.99, 1.0)	0.99 (0.99, 1.00)
Close to major road (ref: yes)					1.48 (0.76, 2.89)	1.41 (0.64, 3.09)
Distance to nearest road (1/miles)					0.15 (0.01, 4.15)	0.49 (0.02, 9.67)

In model 1 for both males and females, the odds of participating in AC increased with increasing neighbourhood deprivation score, with the strongest effect seen in deprivation quintile 5 compared to quintile 1 (adjusted OR:4.24 95%CI 2.41, 7.44 for males and adjusted OR:3.34 95%CI 1.61, 6.94 for females). In fully adjusted models no other social factors were associated with AC behaviour.

Model 2 additionally adjusted for employment-related factors. In females, neighbourhood deprivation remained a significant correlate of AC; however, for males the association was attenuated. Distance to work was the overwhelming correlate of AC. In males, the odds of AC were 88% lower (adjusted OR: 0.12 95%CI: 0.08, 0.17) in those who lived between 1.5 and 4 miles from work compared to those who lived within 1.5 miles. The odds of AC were

For peer review only - http://bmjopen.bmj.com/site/about/guidelines.xhtml

For peer review only

93% lower (adjusted OR: 0.07 95%CI 0.05, 0.11) in females who lived between 1.5 and 4 miles from work with no participants who lived further than 10 miles away reporting AC. In males, no other employment characteristics were associated with AC. In females, there was a suggestion that women whose job involved walking were more likely to use AC (adjust OR: 1.20 95%CI 1.00, 1.43).

Model 3 explored possible environmental correlates of AC behaviour. The only variable associated with AC was population density, in males only. The males who lived in a rural area had a 78% reduced odds of AC compared to those who lived in an urban or fringe area (adjusted OR 0.12 95% CI 0.02, 0.90). No other environmental variables were associated with AC.

DISCUSSION

The overwhelming majority (80%) of adults with T2DM reported commuting by car. AC was reported by 5.5% of the sample, of whom fewer than 1% reported cycling. AC was strongly and consistently associated with higher PA. In fully adjusted models, AC was associated with an additional 73 minutes/week of MVPA for males and 105 minutes/week of MVPA for females, making a substantial contribution to meeting the UK Government recommended levels of 150 minutes/week MVPA [7]. This finding supports previous work by Audrey et al [19] which found levels of PA to be higher in those who walked to work compared to those who did not. Furthermore the effect on MVPA seen in the present study is substantially larger than achieved in a similar population undergoing an intensive PA intervention [8], suggesting that adoption of AC could be an effective intervention for people with T2D.

The importance of sedentary time for both prevention and management of type 2 diabetes has gained research prominence in recent years [32,33], and we found a substantial association between AC and lower sedentary time. In both sexes both AC and public and mixed modes of transport were associated with over an hour less sedentary time per day compared to car use. Since adults with T2DM typically spend less than 3% of the day engaged in MVPA, interventions which focus on reducing sedentary time may be more effective to improve health [34]; re-allocating 30 minutes of prolonged sedentary time to more active pursuits can improve body composition and cholesterol levels in adults with T2DM [35].

The predominant driver of AC was distance to work with few people who lived over 1.5 miles from their workplace choosing AC and those living in rural areas less likely to actively commute. Although distance from home to work is not modifiable, AC could be encouraged by providing more cycleways, by road safety measures to limit traffic speed and improving access to parks and green spaces [36][37]. In addition, public transport to work can

For peer review only - http://bmjopen.bmj.com/site/about/guidelines.xhtml

contribute to PA, though the current study found mixed results therefore the contribution of public transport use to increasing PA warrants further investigation.

This study has some major strengths. UK Biobank provides a large, geographically diverse data source allowing multiple social, environmental and employment related factors to be adjusted for in analyses. The study sample is restricted to adults with T2DM and is the first study of its kind to explore these behaviours on such a scale.

The study also has some important limitations. The analyses were cross-sectional and therefore causality cannot be determined. The small numbers of participants reporting AC did not allow walking and cycling to be examined separately. The relative energy demands of these behaviours differ and previous research has suggested these behaviours are performed by different portions of society [38]. The current study was also unable to distinguish between the different modes of public transport. The observed gender difference suggests there may be differences in the work and travel patterns of males and females in this study population. The average distance from home to work was almost twice as far for males which may explain why more females reported active modes of travel. The study is further limited by self-report measures of PA and sedentary behaviour.

The study sample is predominantly of White British ethnicity which does not reflect the distribution of type 2 diabetes in the UK limiting the generalisability of the findings. The sample is also limited to those who work and therefore can only examine the role of active commuting on PA and sedentary time. The contribution of non-work active travel may be of more interest for people with T2DM who in general are older than the UK average.

In conclusion, AC is associated with increased PA, and reduced sedentary time in people with type 2 diabetes. This may have a beneficial effect on glucose control and could contribute towards reduced treatment costs. Furthermore, AC has the dual benefit of reducing congestion, improving air pollution and contributing to improved psychological outcomes [39]. Therefore, strategies to improve the environment and encourage AC may help to increase population levels of PA and reduce the disease burden associated with type 2 diabetes.

FUNDING

This project was funded by a small project grant from the National Institute for Health Research Bristol Nutrition Biomedical Research Unit. The funding covered the cost of data access only.

AUTHORS CONTRIBUTIONS

For peer review only - http://bmjopen.bmj.com/site/about/guidelines.xhtml

CF, AC and EF made substantial contributions to the conception and design, acquisition of data, or analysis and interpretation of data. All authors drafted the article or revised it critically for important intellectual content. All authors approved the final version of the manuscript to be published.

DATA SHARING STATEMENT

No additional data is available.

COMPETING INTEREST STATEMENT

There are no competing interests to declare by any authors.

REFERENCES

1 Hu FB, Manson JE, Stampfer MJ, *et al.* Diet, lifestyle, and the risk of type 2 diabetes mellitus in women. *N Engl J Med* 2001;**345**:790–7. doi:10.1056/NEJMoa010492

2 Kumari M, Head J, Marmot M, *et al.* Prospective Study of Social and Other Risk Factors for Incidence of Type 2 Diabetes in the Whitehall II Study. *Arch Intern Med* 2004;**164**:1873. doi:10.1001/archinte.164.17.1873

3 WHO (World Health Organisation). Global Report on Diabetes. *WHO* Published Online First: 2016.http://apps.who.int/iris/bitstream/10665/204871/1/9789241565257_eng.pdf?ua=1 (accessed 22 Jul2016).

4 Hex N, Bartlett C, Wright D, *et al.* Estimating the current and future costs of Type 1 and Type 2 diabetes in the UK, including direct health costs and indirect societal and productivity costs. *Diabet Med* 2012;**29**:855–62. doi:10.1111/j.1464-5491.2012.03698.x

5 Mary G, Caroline H, Naomi H, *et al.* Adult obesity and type 2 diabetes. Published Online First: 2014.www.gov.uk/phe (accessed 6 Jul2016).

6 Sigal RJ, Kenny GP, Wasserman DH, *et al.* Physical activity/exercise and type 2 diabetes: a consensus statement from the American Diabetes Association. *Diabetes Care* 2006;**29**:1433–8. doi:10.2337/dc06-9910

7 Chief Medical Officer. Physical activity guidelines for Adults (19-64 years). 2011.

8 Andrews RC, Cooper AR, Montgomery AA, *et al.* Diet or diet plus physical activity versus usual care in patients with newly diagnosed type 2 diabetes: the Early ACTID randomised controlled trial. *Lancet (London, England)* 2011;**378**:129–39. doi:10.1016/S0140-6736(11)60442-X

9 JAKICIC JM, GREGG E, KNOWLER W, *et al.* Activity Patterns of Obese Adults with Type 2 Diabetes in the Look AHEAD Study. *Med Sci Sport Exerc* 2010;**42**:1995–2005. doi:10.1249/MSS.0b013e3181e054f0

10 Grøntved A, Hu FB. Television viewing and risk of type 2 diabetes, cardiovascular disease, and all-cause mortality: a meta-analysis. *JAMA* 2011;**305**:2448–55. doi:10.1001/jama.2011.812

11 Cassidy S, Chau JY, Catt M, *et al.* Cross-sectional study of diet, physical activity, television viewing and sleep duration in 233,110 adults from the UK Biobank; the

For peer review only - http://bmjopen.bmj.com/site/about/guidelines.xhtml

behavioural phenotype of cardiovascular disease and type 2 diabetes. *BMJ Open* 2016;**6**:e010038. doi:10.1136/bmjopen-2015-010038

12 Physical activity: walking and cycling | Guidance and guidelines | NICE.

13 FPH. Take action on active travel. Fac. Public Heal. 2008.http://www.fph.org.uk/uploads/Take_action_on_active_travel.pdf (accessed 15 May2016).

14 Laverty research fellow AA, Hérick de Sá student T, Monteiro professor CA. Getting sedentary people moving through active travel. *J Epidemiol Community Heal* 2012;**66**:176–80. doi:10.1136/bmj.h725

15 Goodman A, Hamer M, Chida Y, *et al.* Walking, Cycling and Driving to Work in the English and Welsh 2011 Census: Trends, Socio-Economic Patterning and Relevance to Travel Behaviour in General. *PLoS One* 2013;**8**:e71790. doi:10.1371/journal.pone.0071790

16 Flint E, Cummins S, Sacker A. Associations between active commuting, body fat, and body mass index: population based, cross sectional study in the United Kingdom. BMJ (Clinical Res. ed). 2014. doi:10.1136/bmj.g4887 <http://dx.doi.org/10.1136/bmj.g4887>

17 Flint E, Cummins S. Does active commuting protect against obesity in mid-life? Cross-sectional, observational evidence from UK Biobank. *Lancet* Published Online First: 2015. doi:10.1016/S0140-6736(15)00846-6

18 Laverty AA, Mindell JS, Webb EA, *et al.* Active Travel to Work and Cardiovascular Risk Factors in the United Kingdom. *Am J Prev Med* 2013;**45**:282–8. doi:10.1016/j.amepre.2013.04.012

19 Audrey S, Procter S, Cooper AR, *et al.* The contribution of walking to work to adult physical activity levels: a cross sectional study. *Int J Behav Nutr Phys Act* 2014;**11**:37. doi:10.1186/1479-5868-11-37

20 Flint E, Webb E, Cummins S. Change in commute mode and body-mass index: prospective, longitudinal evidence from UK Biobank. *Lancet Public Heal* 2016;**1**:e46–55. doi:10.1016/S2468-2667(16)30006-8

21 Mytton OT, Panter J, Ogilvie D. Longitudinal associations of active commuting with body mass index. *Prev Med (Baltim)* 2016;**90**:1–7. doi:10.1016/j.ypmed.2016.06.014

22 Stewart G, Anokye NK, Pokhrel S. What interventions increase commuter cycling? A systematic review. *BMJ Open* 2015;**5**:e007945. doi:10.1136/bmjopen-2015-007945

23 Frank LD, Saelens BE, Powell KE, *et al.* Stepping towards causation: Do built environments or neighborhood and travel preferences explain physical activity, driving, and obesity? *Soc Sci Med* 2007;**65**:1898–914. doi:10.1016/j.socscimed.2007.05.053

24 Allen NE, Sudlow C, Peakman T, *et al.* UK biobank data: come and get it. *Sci Transl Med* 2014;**6**:224ed4. doi:10.1126/scitranslmed.3008601

25 Biobank U. Rationale, design and development of a large-scale prospective resource. UK Biobank. 2007.http://www.ukbiobank.ac.uk/resources/

26 Craig CL, Marshall AL, Sjöström M, *et al.* International physical activity questionnaire: 12-country reliability and validity. *Med Sci Sports Exerc* 2003;**35**:1381–95. doi:10.1249/01.MSS.0000078924.61453.FB

27 IPAQ. Guidelines for Data Processing and Analysis of the International Physical

For peer review only - http://bmjopen.bmj.com/site/about/guidelines.xhtml

Activity Questionnaire (IPAQ) - Short Form,.

28 Small area health statistics unit. www.sashu.org

29 BioSHaRE EU Determinants of Health Project. http://bioshare.eu

30 Eeftens M, Beelen R, Hoogh K de, *et al.* Development of Land Use Regression Models for PM2.5, PM2.5 Absorbance, PM10 and PMcoarse in 20 European Study Areas; Results of the ESCAPE Project. 2012.

31 StataCorp 2015. Stata statistical software: Release 14.

32 Brocklebank LA, Falconer CL, Page AS, *et al.* Accelerometer-measured sedentary time and cardiometabolic biomarkers: A systematic review. *Prev Med (Baltim)* Published Online First: 2015. doi:10.1016/j.ypmed.2015.04.013

33 Owen N, Sparling PB, Healy GN, *et al.* Sedentary behavior: emerging evidence for a new health risk. *Mayo Clin Proc* 2010;**85**:1138–41. doi:10.4065/mcp.2010.0444

34 Falconer CL, Cooper AR, Walhin JP, *et al.* Sedentary time and markers of inflammation in people with newly diagnosed type 2 diabetes. *Nutr Metab Cardiovasc Dis* 2014;**24**:956–62. doi:10.1016/j.numecd.2014.03.009

35 Falconer CL, Page AS, Andrews RC, *et al.* The Potential Impact of Displacing Sedentary Time in Adults with Type 2 Diabetes. *Med Sci Sports Exerc* 2015;**47**:2070–5. doi:10.1249/MSS.0000000000000651

36 Mackenbach JD, Rutter H, Compernolle S, *et al.* Obesogenic environments: a systematic review of the association between the physical environment and adult weight status, the SPOTLIGHT project. *BMC Public Health* 2014;**14**:233. doi:10.1186/1471-2458-14-233

37 Tomlinson P, Hewitt S, Blackshaw N. Joining up health and planning: how Joint Strategic Needs Assessment (JSNA) can inform health and wellbeing strategies and spatial planning. *Perspect Public Health* 2013;**133**:254–62. doi:10.1177/1757913913488331

38 Mytton OT, Panter J, Ogilvie D. Longitudinal associations of active commuting with wellbeing and sickness absence. *Prev Med (Baltim)* 2016;**84**:19–26. doi:10.1016/j.ypmed.2015.12.010

39 Saunders LE, Green JM, Petticrew MP, *et al.* What are the health benefits of active travel? A systematic review of trials and cohort studies. *PLoS One* 2013;**8**:e69912. doi:10.1371/journal.pone.0069912

For peer review only

For peer review only - http://bmjopen.bmj.com/site/about/guidelines.xhtml

For peer review only

For peer review only - http://bmjopen.bmj.com/site/about/guidelines.xhtml

For peer review only

For peer review only - http://bmjopen.bmj.com/site/about/guidelines.xhtml

STROBE Statement—checklist of items that should be included in reports of observational studies

	Item No	Recommendation	Page number
**Title and abstract**	1	(*a*) Indicate the study's design with a commonly used term in the title or the abstract	1
		(*b*) Provide in the abstract an informative and balanced summary of what was done and what was found	2
**Introduction**			
Background/rationale	2	Explain the scientific background and rationale for the investigation being reported	4-5
Objectives	3	State specific objectives, including any prespecified hypotheses	5
**Methods**			
Study design	4	Present key elements of study design early in the paper	6
Setting	5	Describe the setting, locations, and relevant dates, including periods of recruitment, exposure, follow-up, and data collection	6
Participants	6	(*a*) *Cohort study*—Give the eligibility criteria, and the sources and methods of selection of participants. Describe methods of follow-up<br>*Case-control study*—Give the eligibility criteria, and the sources and methods of case ascertainment and control selection. Give the rationale for the choice of cases and controls<br>*Cross-sectional study*—Give the eligibility criteria, and the sources and methods of selection of participants	6
		(*b*) *Cohort study*—For matched studies, give matching criteria and number of exposed and unexposed<br>*Case-control study*—For matched studies, give matching criteria and the number of controls per case	
Variables	7	Clearly define all outcomes, exposures, predictors, potential confounders, and effect modifiers. Give diagnostic criteria, if applicable	6-7
Data sources/ measurement	8*	For each variable of interest, give sources of data and details of methods of assessment (measurement). Describe comparability of assessment methods if there is more than one group	6-7
Bias	9	Describe any efforts to address potential sources of bias	7
Study size	10	Explain how the study size was arrived at	NA
Quantitative variables	11	Explain how quantitative variables were handled in the analyses. If applicable, describe which groupings were chosen and why	8
Statistical methods	12	(*a*) Describe all statistical methods, including those used to control for confounding	8
		(*b*) Describe any methods used to examine subgroups and interactions	88
		(*c*) Explain how missing data were addressed	
		(*d*) *Cohort study*—If applicable, explain how loss to follow-up was addressed<br>*Case-control study*—If applicable, explain how matching of cases and controls was addressed<br>*Cross-sectional study*—If applicable, describe analytical methods taking account of sampling strategy	8
		(*e*) Describe any sensitivity analyses	8

Continued on next page

For peer review only

**Results**			
Participants	13*	(a) Report numbers of individuals at each stage of study—eg numbers potentially eligible, examined for eligibility, confirmed eligible, included in the study, completing follow-up, and analysed	
		(b) Give reasons for non-participation at each stage	
		(c) Consider use of a flow diagram	
Descriptive data	14*	(a) Give characteristics of study participants (eg demographic, clinical, social) and information on exposures and potential confounders	8
		(b) Indicate number of participants with missing data for each variable of interest	
		(c) *Cohort study*—Summarise follow-up time (eg, average and total amount)	
Outcome data	15*	*Cohort study*—Report numbers of outcome events or summary measures over time	8
		Case-control study—Report numbers in each exposure category, or summary measures of exposure	
		Cross-sectional study—Report numbers of outcome events or summary measures	
Main results	16	(*a*) Give unadjusted estimates and, if applicable, confounder-adjusted estimates and their precision (eg, 95% confidence interval). Make clear which confounders were adjusted for and why they were included	8-10
		(*b*) Report category boundaries when continuous variables were categorized	
		(*c*) If relevant, consider translating estimates of relative risk into absolute risk for a meaningful time period	
Other analyses	17	Report other analyses done—eg analyses of subgroups and interactions, and sensitivity analyses	8-10
**Discussion**			
Key results	18	Summarise key results with reference to study objectives	10-11
Limitations	19	Discuss limitations of the study, taking into account sources of potential bias or imprecision. Discuss both direction and magnitude of any potential bias	12
Interpretation	20	Give a cautious overall interpretation of results considering objectives, limitations, multiplicity of analyses, results from similar studies, and other relevant evidence	12
Generalisability	21	Discuss the generalisability (external validity) of the study results	12
**Other information**			
Funding	22	Give the source of funding and the role of the funders for the present study and, if applicable, for the original study on which the present article is based	13

*Give information separately for cases and controls in case-control studies and, if applicable, for exposed and unexposed groups in cohort and cross-sectional studies.

Note: An Explanation and Elaboration article discusses each checklist item and gives methodological background and published examples of transparent reporting. The STROBE checklist is best used in conjunction with this article (freely available on the Web sites of PLoS Medicine at http://www.plosmedicine.org/, Annals of Internal Medicine at http://www.annals.org/, and Epidemiology at http://www.epidem.com/). Information on the STROBE Initiative is available at www.strobe-statement.org.

For peer review only

For peer review only - http://bmjopen.bmj.com/site/about/guidelines.xhtml

BMJ Open

Patterns and correlates of active commuting in adults with type 2 diabetes: cross-sectional evidence from UK Biobank

Journal:	*BMJ Open*
Manuscript ID	bmjopen-2017-017132.R1
Article Type:	Research
Date Submitted by the Author:	31-May-2017
Complete List of Authors:	Falconer, Catherine; Gloucestershire Hospitals NHS Foundation Trust, ; University of Bristol, NIHR Bristol Nutrition Biomedical Research Unit<br>Cooper, Ashley; University of Bristol<br>Flint, Ellen; London School of Hygiene and Tropical Medicine, Social and Environmental Health Research
**Primary Subject Heading**:	Public health
Secondary Subject Heading:	Sports and exercise medicine
Keywords:	General diabetes < DIABETES & ENDOCRINOLOGY, EPIDEMIOLOGY, PREVENTIVE MEDICINE, PUBLIC HEALTH

SCHOLARONE™
Manuscripts

Title Page

Patterns and correlates of active commuting in adults with type 2 diabetes: cross-sectional evidence from UK Biobank

Falconer, Catherine L[1,2]; Cooper, Ashley R[2,3]; Flint, Ellen[4]

[1]NHS Gloucestershire Foundation Trust,

[2]NIHR Bristol Nutrition Biomedical Research Unit, University of Bristol

[3]Centre for Exercise, Nutrition and Health Sciences, University of Bristol

[4] Department of Social and Environmental Health Research, London School of Hygiene and Tropical Medicine

For peer review only

Word Count: 2,920
Abstract: 272
Number of tables: 3
Number of figures: 0

Corresponding author:

Professor Ashley Cooper
Bristol Nutrition BRU
Level 3, University Hospitals Research and Education Centre,
Upper Maudlin Street,
Bristol,
BS2 8AE
Email: Ashley.cooper@bristol.ac.uk

For peer review only - http://bmjopen.bmj.com/site/about/guidelines.xhtml

ABSTRACT

Objectives To describe the active commuting patterns of adults with type 2 diabetes and how these relate to physical activity and sedentary behaviour in UK Biobank. Social and environmental correlates of active commuting will also be explored.

Design Cross-sectional analysis of a cohort study

Settings Population cohort of over 500,000 people recruited from 22 centres across the United Kingdom. Participants aged between 37 and 73 years were recruited between 2006 and 2010.

Participants 6,896 participants with a self-reported type 2 diabetes diagnosis who reported commuting to work and had complete covariate data were included in the analysis.

Exposure measures Exposure measures were active commuting (AC) to work, measured as usual mode of transport.

Outcome measures Outcome measures were weekly minutes of moderate-to-vigorous physical activity (MVPA), and hours/day of sedentary time, and participation in active travel.

Results AC was reported by 5.5% of participants with the overwhelming majority using the car to commute (80%). AC was associated with an additional 73 (95%CI 10.8, 134.9) and 105 (95%CI 41.7, 167.2) weekly minutes of MVPA for males and females respectively. AC was associated with reduced sedentary time (β-1.1 95%CI -1.6, -0.7 hours/day for males and β-0.8 95%CI -1.2, -0.3 hours/day for females). Deprivation and distance from home to work were identified as correlates of AC behaviour.

Conclusions Rates of active commuting are very low in adults with type 2 diabetes. However, active commuting offers a potentially sustainable solution to increasing physical activity and reducing sedentary behaviour. Therefore, strategies to improve the environment and encourage active commuting may help to increase population levels of physical activity and reduce the disease burden associated with type 2 diabetes.

For peer review only - http://bmjopen.bmj.com/site/about/guidelines.xhtml

Article summary

Strengths and limitations of this study

This study uses the UK Biobank to examine the active commuting behaviours of adults with type 2 diabetes. UK Biobank is a large, geographically diverse cohort which provides the largest exploration of active commuting in this population to date.

UK Biobank provided the opportunity to be able to explore a multitude of employment, social and environmental factors which may contribute to active commuting.

We were unable to distinguish between walking and cycling behaviour, and also were unable to explore different type of public transport modes for example bus or train.

The analysis used in this study is cross-sectional and therefore we cannot infer causality.

This study is further limited by the use of self-report measures of physical activity and sedentary time.

For peer review only

For peer review only - http://bmjopen.bmj.com/site/about/guidelines.xhtml

INTRODUCTION

Type 2 diabetes mellitus (T2DM) is a disease associated with unhealthy lifestyle behaviours, such as poor diet and physical inactivity [1] and displays a social gradient [2]. The worldwide age-standardised prevalence of T2DM has doubled to 8.5% since 1980 [3]. In the UK the prevalence is 6% and the ongoing treatment and management burden of T2DM and its associated complications places a considerable burden on the National Health Service [4,5].

The role of physical activity (PA) in the prevention and management of T2DM is well understood [6]. However, fewer adults with T2DM achieve UK Government recommendations of at least 150 minutes moderate-to-vigorous physical activity (MVPA) per week [7] and many interventions fail to achieve increases in PA sufficient to confer metabolic benefits [7–9]. Sedentary behaviour is also an independent risk factor for the development of T2DM [10]. Cross-sectional evidence from UK Biobank suggests that adults with T2DM are characterised by a phenotype of unhealthy behaviours including prolonged sedentary time, physical inactivity and poor sleep, suggesting that these behaviours should be targeted in parallel [11].

The role of active commuting in increasing PA and controlling excessive weight gain has been recognised and endorsed by the National Institute for Health and Care Excellence (NICE) [12]. Active commuting has the potential to increase individuals PA and reduce sedentary time, and more widely to lower levels of traffic congestion and noise and air pollution [13], and can be sustainably incorporated into daily living [14]. However, rates of AC are low in the UK. In the 2011 census, of the 58% of adults who responded to the question 'how do you usually travel to work?, 67% reported private motorised transport as the principle mode of transport,17.8% public transport, 10.9% walking and 3.1% commuting by bicycle [15].

There is increasing evidence of an association between AC and measures of obesity [16,17]. In the UK Understanding Society cohort, active commuting has been shown to be predictive of lower BMI and body fat percentage, and a lower adjusted odds of reporting a diabetes diagnosis compared to car users [16][18]. Similarly in UK Biobank, AC was significantly and independently associated with lower BMI and body fat percentage [17]. The protective effect of AC is likely to be mediated through increased PA as demonstrated in a UK study which used accelerometry and GPS receivers to show that walking to work contributed 47.3% of daily MVPA [19].

Causal evidence from longitudinal or experimental studies is rare. A longitudinal analysis of the UK Biobank cohort looked at changes in BMI in response to changes in commuting mode over a 4 year follow up. Individuals who transitioned from car commuting at baseline to

For peer review only - http://bmjopen.bmj.com/site/about/guidelines.xhtml

active or public transportation modes at follow up had a decrease in BMI of -0.30kg/m^2 (95%CI -0.47, -0.13) [20].

Promoting AC may help to increase population levels of activity; however, there is a need to understand the correlates of AC to develop appropriate interventions. Some key determinants of commuter cycling and walking behaviour have been identified as high population density, dedicated cycling paths, short trip distances and good traffic and road conditions, such as the presence of traffic calming measures [21][22]. Most work to date has been performed in healthy adults and there is a need to understand how these findings relate to populations with T2DM who are characterised by obesity and physical inactivity.

The primary aim of this work is to describe the AC patterns of adults with T2DM in UK Biobank. Specifically, the study explores how AC is associated with PA and sedentary time in this population (objective 1). The study will also aim to identify social and environmental factors (age, social class and traffic density) that may predict participation in AC (objective 2).

METHODS

Study population

Baseline data from the UK Biobank study were used (project 19307). UK Biobank is a large population study which aims to examine how the environment, lifestyle and genes interact to affect health [23]. Full recruitment procedures are described in detail elsewhere, [24] and briefly summarised below.

Figure 1. Flow chart demonstrating how the population sample was identified.

Between 2006 and 2010, recruitment invitations were sent to 9.2 million people from 22 assessment centres around the UK to obtain a sample of 502,565 adults aged 37-73 years [11]. Following recruitment, participants provided written, informed consent and attended an assessment centre for data collection. A touchscreen questionnaire covered socio-demographics, occupation, lifestyle, early exposures, cognitive function, family history and medical history. Participants also underwent a verbal interview, physical measures and provided a blood/urine sample. All data were anonymised. The UK Biobank Access Subcommittee granted approval for the data access under a Material Transfer Agreement with the London School of Hygiene and Tropical Medicine who provided ethical approval for this project (LSHTM Ethics reference: 10838).

For peer review only - http://bmjopen.bmj.com/site/about/guidelines.xhtml

Study sample

Participants who reported having diabetes or T2DM were selected (n=26,408). We excluded anyone <35 years at diagnosis of diabetes and who reported taking insulin within the first year of diagnosis to reduce the likelihood of type 1 or monogenic forms of diabetes [11], (n=20,143). Only participants who reported commuting behaviours and had complete covariate data were included in the analysis (n=6,896).

Active commuting measures

In response to the question "what types of transport do you use to get to and from work?", participants could select one or more of the following options: car/motor vehicle; walk; public transport; cycle. In order to answer the two study objectives, two exposure variables were derived: 1. a three-category exposure variable; (1) *car* or *car and public transport*; (2) *public transport* and *mixed* (car and walk/cycle; public transport and walk/cycle); (3) AC: *walk* or *cycle* only and 2. a binary variable ((1) No active commuting; (2) Active commuting (AC: walk or cycle only)). Only participants who exclusively reported walking or cycling behaviour were included in the active commuting categories.

Physical activity

PA was assessed using questions adapted from the validated short form of the International Physical Activity Questionnaire (IPAQ) [25]. The questions asked participants to self-report the frequency, duration and intensity of walking, moderate and vigorous activity. From these questions, we derived three measures of physical activity. A variable for total physical activity (total MET-minutes/week) was derived by weighting time spent in vigorous, moderate and walking activity by the relevant energy expended for each activity to produce metabolic equivalents, MET-minutes per week of PA [26]. Duration of MVPA was derived as the sum of reported duration and frequency of moderate and vigorous activity. From this, a variable for 'attainment of physical activity guidelines' (150 minutes' moderate PA or 75 minutes of vigorous activity per week) was derived[7].

Sedentary behaviour

Self-reported television viewing, computer use and driving behaviour were collected from the touchscreen questionnaire. An estimate of average total sedentary time was derived by summing the daily reported duration of television viewing, computer use and driving. Total sedentary time is expressed as hours per day.

Environmental correlates

Local environmental exposure variables were collated by the Small Area Health Statistics Unit [27] as part of the BioSHaRE-EU Environmental Determinants of Health Project [28]. Air

For peer review only - http://bmjopen.bmj.com/site/about/guidelines.xhtml

pollution estimates were modelled for the year 2010 using a Land Use Regression (LUR) model developed as part of the European Study of Cohorts for Air Pollution Effects (ESCAPE) [29]. ESCAPE estimates of particulate matter (PM) are valid up to 400km from the monitoring area, which in this case was Greater London. Therefore, participants who have an address more than 400km from this site were not assigned a value for PM2.5-10. Traffic variables, including inverse distance to a major road and traffic density, were also calculated using the LUR model, based on Eurostreets (v3.1) digital road network for the year 2008. Traffic intensity is measured as the average total number of motor vehicles per 24 hours on the nearest road.

Covariates

A range of confounding variables were considered. Socioeconomic covariates were; Townsend Deprivation Index of residential area, annual gross household income, highest educational qualification. Further demographic covariates were: age (years); sex; ethnicity, census-derived residential area population density classification (urban/fringe, rural). The potential confounding effects of occupational physical activity were considered: job involves standing/walking; job involves heavy manual/physical work, shift work, non-work related transport modes (active modes: yes/no). Health covariates were: poor self-rated health (yes/no). Commute frequency (number of outward journeys per week, truncated at 10) and distance between home and workplace (reported in miles) were included as covariates in the model.

Statistical analyses

Descriptive analyses were undertaken to describe the commuting patterns of the population, the prevalence of PA and sedentary behaviours and the patterning of hypothesised confounding variables. Continuous variables are displayed as mean and standard deviation and frequencies are used for categorical variables.

To address objective 1, multivariate linear regression was used to describe the association between AC and each of the two outcome variables (PA and sedentary time) adjusting for potential confounding variables in two step-wise series of three nested models. The first model presents the unadjusted association between the exposure (commuting mode) and outcome of interest. The second model adjusts for demographic and socio-economic factors. The third model additionally adjusts for employment-related confounders such as distance from home to work.

To address objective 2, multivariate logistic regression modelling was used to explore potential socio-demographic, environmental and employment-related correlates of AC behaviour. The model used was adjusted for all included variables. All analyses controlled

For peer review only - http://bmjopen.bmj.com/site/about/guidelines.xhtml

for geographical clustering by assessment centre, and potential interactions by gender, ethnicity and household income were explored. For these analyses, the outcome was expressed as a binary categorical variable ((1) not active commuting, (2) active commuting). All analyses were conducted using Stata v14 [30].

RESULTS

The final sample was 6,896 participants with T2DM who reported commuting behaviour and provided complete data on all covariates (Table I).

Table 1. Demographic, commuting behaviour, employment, physical activity and environmental characteristics of the study population

Variable	Males (n=4,473)	Females (n=2,423)	Total (n=6,896)
**Demographics**			
Age (years)	56.7 ± 6.5	54.53 ± 6.6	55.9 ± 6.6
Ethnicity (%)			
White British	81.8	78.2	80.5
Other white	5.3	6.5	5.7
South Asian	6.2	4.2	5.5
Black Caribbean	1.3	3.9	2.2
Black African	1.8	2.1	1.9
Chinese	0.3	0.6	0.4
Mixed ethnicity	0.6	0.8	0.7
Other ethnicity	2.7	3.8	3.1
Townsend Index of deprivation(quintiles) (%)			
1(most deprived)	16.1	14.1	15.4
2	17.2	15.8	16.7
3	19.6	20.9	20.1
4	21.2	22.1	21.8
5 (least deprived)	25.5	27.1	26.0
Highest Educational Qualification (%)			
University	29.6	30.8	30.0
A Levels/AS Levels	11.2	11.2	11.4
GCSE/O Levels	20.5	25.0	22.1
CSE's or equivalent	6.4	7.9	6.9
NVQ or HND or HNC	11.5	7.0	9.9
Other professional qualifications	5.32	6.2	5.6
None	15.6	11.6	14.2
Average household income (%)			
<£18,000	12.2	20.5	15.1
£18,000 to £30,999	27.4	28.0	27.6
£31,000 to £51,999	30.9	29.3	30.4
£52,000 to £100,000	23.8	18.6	22.0
>£100,000	5.7	3.5	4.9
Poor self-rated health (%)	9.9	9.0	9.6
**Employment**			
Job involves heavy lifting (%)			
Never/rarely	59.8	65.8	61.9
Sometimes	25.0	22.5	24.1
Usually	8.1	5.7	7.2
Always	7.1	6.0	6.7
Job involves walking(%)			
Never/rarely	31.3	32.8	31.8
Sometimes	32.7	31.2	32.2
Usually	16.1	13.8	15.3
Always	19.9	22.2	20.7
Shift work	7.7	5.9	7.1

For peer review only - http://bmjopen.bmj.com/site/about/guidelines.xhtml

For peer review only

**Commuting behaviour**			
Commute frequency (no. outward journeys/week)	4.6 ± 2.1	4.3 ± 1.8	4.5 ± 2.0
Distance from home to work (miles)	14.9 ± 28.4	7.5 ± 9.9	12.3 ± 24.0
Commuting mode (%)			
Motorised travel	83.2	75.3	80.4
Motorised travel plus active travel	12.3	17.5	14.1
Active commuting	4.5	7.2	5.4
Non work active travel	40.4	40.2	40.3
**Physical activity and sedentary time**			
Daily sedentary time (hours)	6.0 ± 3.1	4.9 ± 2.7	5.6 ± 3.0
Total weekly MVPA[1] (minutes)	263.9 ± 446.4	214.9 ± 383.4	246.6 ± 425.9
Total weekly MET[2]-minutes	2373.3 ± 3099.8	2014.7 ± 2854.7	2245.5 ± 3106.2
Achieves PA guidelines[3] (%)	54.3	53.8	54.1
**Environmental characteristics**			
Close to major road (%)	8.0	8.2	8.1
Population density (urban/fringe) (%)	94.5	95.7	94.9
Inverse distance to nearest road (1/m)	0.05 (0.05, 0.05)	0.05 (0.05, 0.05)	0.05 (0.05, 0.05)
Air pollution PM2.5-10 (micro-g/m^3)	6.5 ± 0.9	6.5 ± 0.9	6.5 ± 0.9
Traffic density (vehicles/day)	1699.4 ± 5663.6	1716.2 ± 6057.0	1698.6 ± 5663.6

[1]MVPA, Moderate to vigorous physical activity; [2]MET, metabolic equivalents (1MET=1kcal/kg/hr); [3]PA guidelines, physical activity guidelines (150 minutes MVPA/week or 75 minutes vigorous activity/week)[7]

Car travel was reported by 83.2% of male and 75.2% of female participants, with walking or cycling exclusively reported by 4.5% of males and 7.2% of females. Females spent less time sedentary than males (4.9 ± 2.7 hours/day versus 6.0 ± 3.1 hours/day), while males reported more weekly MVPA (263.9 ± 446.4 minutes/week versus 214.9 ± 383.4 minutes/week).

The results of analyses to investigate the association between commuting mode, PA and sedentary behaviour (objective 1) are displayed in table 2. A significant interaction for gender was found so stratified analyses are presented (p<0.1).

For peer review only

For peer review only - http://bmjopen.bmj.com/site/about/guidelines.xhtml

Table 2. Results of linear regression modelling to investigate the associations between commuting behaviour, physical activity and sedentary time, stratified by gender.

	MVPA (minutes/week)[1]		Total MET-minutes/week		Total sedentary time (hours/day)	
	**Males β (95% CI)**	**Females β (95% CI)**	**Males β (95% CI)**	**Females β (95% CI)**	**Males β (95% CI)**	**Females β (95% CI)**
**Model 1**						
Motorised travel	0.0	0.0	0.0	0.0	0.0	0.0
(reference)	-49.9 (-89.8, -10.1)	12.5 (-27.9, 52.8)	93.0 (-381.0, 193.0)	312.3 (12.3, 612.4)	-2.0 (-2.3, -1.7)	-1.0 (-1.3, 0.7)
MT/AC[2]	86.4 (23.1, 149.7)	156.2 (97.0, 215.4)	777.0 (320.6, 1233.3)	1287.3 (847.3, 1727.4)	-1.3 (-1.8, -0.9)	-1.0 (-1.4, -0.6)
Active commuting						
**Model 2[3]**						
Car (reference)	0.0	0.0	0.0	0.0	0.0	0.0
Mixed/PT	-57.4 (-98.6, -16.2)	-3.0 (-45.9, 40.1)	-156.1 (-452.4, 140.3)	155.2 (-163.4, 473.9)	-2.2 (-2.4, -1.9)	-1.2 (-1.5, -0.9)
Active commuting	74.6 (10.9, 138.4)	145.3 (85.5, 205.2)	665.5 (207.0, 1124.1)	1149.4 (706.3, 1592.5)	-1.5 (-1.9, -1.0)	-1.1 (-1.5, -0.7)
**Model 3[4]**						
Car (reference)	0.0	0.0	0.0	0.0	0.0	0.0
Mixed/PT	-29.6 (-70.6, 11.4)	11.5 (-35.3, 58.3)	47.0 (-240.9, 334.9)	290.8 (-46.0, 627.6)	-2.2 (-2.5, -1.9)	-1.1 (-1.5, -0.8)
Active commuting	72.9 (10.8, 134.9)	104.5 (41.7, 167.2)	586.6 (150.9, 1022.3)	722.3 (270.3, 1174.1)	-1.1 (-1.6, -0.7)	-0.8 (-1.2, -0.3)

[1]MVPA: Moderate to vigorous physical activity; [2]MT/AC: motorised travel and active commuting; [3]Model is adjusted for age, ethnicity, average household income, Townsend deprivation score, assessment centre, poor health; [4]additionally adjusted for distance from home to work, commute frequency, population density, non-work active travel and job involves walking.

For peer review only

For peer review only - http://bmjopen.bmj.com/site/about/guidelines.xhtml

In all models, AC was associated with higher MVPA. In fully adjusted models, compared to motorised travel, AC was associated with an additional 72.9 (95%CI 10.8, 134.9) minutes/week of MVPA for males and 104.5 (95%CI 41.7, 167.2) minutes/week for females. The association with motorised travel and active commuting was less clear. In males, there was a suggestion that motorised travel and active cmmuting were associated with less MVPA compared to motorised travel alone. This association was attenuated once environmental factors were included in the model (adjusted β 29.6; 95%CI -70.6, 11.4).

AC was also consistently associated with increased total PA in both genders, an effect which survived adjustment for all confounding factors (β 586.6; 95%CI 150.9, 1022.3 for males and β 722.3; 95%CI 270.3, 1174.1 for females). In unadjusted models, motorised travel and active commuting were associated with increased total PA for females only; however, this was attenuated once employment and demographic factors were included in the model.

In both males and females, and in all models, consistent associations were seen between commuting mode and total sedentary time. In fully adjusted models, compared to male motorised travellers, AC was associated with 1.1 (95%CI -1.6, -0.7) fewer hours/day sedentary while mixed motorised travel and active commuting modes were associated with 2.2 (95%CI -2.5, -1.9) fewer sedentary hours/day. A similar effect was seen in females with AC associated with 0.8 (95%CI -1.2, -0.3) fewer sedentary hours/day and mixed motorised travel and active commuting were associated with 1.1 (95%CI -1.5, -0.8) fewer sedentary hours/day compared to car users.

The results of multivariate logistic regression performed to identify social, employment and environmental correlates of AC behaviour (objective 2) are displayed in Table 3. There was evidence of a significant interaction for gender so stratified results are presented.

Table 3. Results of logistic regression modelling to identify potential social, employment and environmental correlates of active travel.

	**Model 1 Individual factors**		**Model 2 Employment factors**		**Model 3 Environmental factors**	
	**Males OR (95% CI)**	**Females OR (95% CI)**	**Males OR (95% CI)**	**Females OR (95% CI)**	**Males OR (95% CI)**	**Females OR (95% CI)**
**Social factors**						
Age (years)	1.0 (0.9, 1.0)	0.98 (0.95, 1.00)	0.98 (0.96, 1.00)	0.97 (0.94, 0.99)	1.01 (0.98, 1.03)	0.97 (0.95, 0.99)
Townsend Deprivation quintile	1.0	1.0	1.0	1.0	1.0	1.0
1 (ref) 2	1.13 (0.58, 2.22)	2.09 (0.93, 4.71)	0.88 (0.42, 1.84)	2.76 (1.10, 6.92)	1.19 (0.60, 2.37)	1.99 (0.88, 4.50)
3 4	1.90 (1.03, 3.49)	3.00 (1.42, 6.37)	1.35 (0.69, 2.64)	2.54 (1.10, 5.90)	1.80 (0.96, 3.37)	2.69 (1.26, 5.75)
5	2.04 (1.12, 3.71)	3.25 (1.55, 6.83)	1.49 (0.77, 2.88)	2.60 (1.14, 5.95)	1.79 (0.96, 3.34)	2.92 (1.38, 6.19)
	4.24 (2.41,	3.34 (1.61,	2.28 (1.23,	3.07 (1.36,	3.32 (1.84,	3.08 (1.47,

For peer review only

For peer review only - http://bmjopen.bmj.com/site/about/guidelines.xhtml

	7.44)	6.94)	4.21)	6.94)	6.01)	6.48)
Average household income (£)						
<£18,000 (ref)	1.0	1.0	1.0	1.0	1.0	1.0
£18,000 to £30,999	0.68 (0.44, 1.04)	0.72 (0.48, 1.09)	0.97 (0.59, 1.59)	1.25 (0.77, 2.04)	0.61 (0.39, 0.97)	0.76 (0.50, 1.16)
£31,000 to £51,999	0.69 (0.44, 1.07)	0.39 (0.24, 0.62)	1.23 (0.74, 2.04)	0.94 (0.53, 1.65)	0.65 (0.42, 1.03)	0.41 (0.25, 0.67)
£52,000 to £100,000	0.85 (0.53, 1.37)	0.35 (0.20, 0.62)	1.34 (0.77, 2.33)	0.88 (0.45, 1.74)	0.80 (0.49, 1.32)	0.37 (0.21, 0.67)
>£100,000	1.10 (0.58, 2.11)	0.90 (0.40, 2.01)	1.27 (0.59, 2.79)	4.27 (1.47, 12.40)	0.90 (0.43, 1.89)	0.75 (0.30, 1.87)
Assessment centre	0.97 (0.95, 1.00)	1.00 (0.98, 1.03)	0.98 (0.95, 1.01)	1.02 (0.98, 1.05)	0.98 (0.95, 1.00)	1.00 (0.97, 1.03)
Poor health	0.70 (0.41, 1.18)	0.83 (0.47, 1.46)	0.56 (0.31, 1.00)	1.04 (0.53, 2.04)	0.62 (0.35, 1.11)	0.89 (0.50, 1.56)
**Employment characteristics**						
Distance from home to work (miles)						
<1.5 (ref)			1.0	1.0		
1.5-4			0.12 (0.08, 0.17)	0.07 (0.05, 0.11)		
4-10			0.01 (0.01, 0.03)	0.01 (0.05, 0.03)		
>10			0.002 (0.001, 0.01)	0.00		
Commute frequency			0.95 (0.86, 1.05)	1.01 (0.91, 1.14)		
Job involves walking			0.97 (0.83, 1.13)	1.20 (1.00, 1.43)		
Shift work			0.85 (0.43, 1.67)	0.76 (0.31, 1.88)		
**Environmental characteristics**						
Population density (ref: urban)					0.12 (0.02, 0.90)	0.39 (0.09, 1.62)
Air pollution (PM10 microg/m3)					1.01 (0.84, 1.21)	0.89 (0.72, 1.09)
Traffic density (no. cars)					1.0 (0.99, 1.0)	0.99 (0.99, 1.00)
Close to major road (ref: yes)					1.48 (0.76, 2.89)	1.41 (0.64, 3.09)
Distance to nearest road (1/miles)					0.15 (0.01, 4.15)	0.49 (0.02, 9.67)

In model 1 for both males and females, the odds of participating in AC increased with increasing neighbourhood deprivation score, with the strongest effect seen in deprivation quintile 5 compared to quintile 1 (adjusted OR:4.24 95%CI 2.41, 7.44 for males and adjusted OR:3.34 95%CI 1.61, 6.94 for females). In fully adjusted models no other social factors were associated with AC behaviour.

Model 2 additionally adjusted for employment-related factors. In females, neighbourhood deprivation remained a significant correlate of AC; however, for males the association was attenuated. Distance to work was the overwhelming correlate of AC. In males, the odds of AC were 88% lower (adjusted OR: 0.12 95%CI: 0.08, 0.17) in those who lived between 1.5

For peer review only - http://bmjopen.bmj.com/site/about/guidelines.xhtml

For peer review only

and 4 miles from work compared to those who lived within 1.5 miles. The odds of AC were 93% lower (adjusted OR: 0.07 95%CI 0.05, 0.11) in females who lived between 1.5 and 4 miles from work with no participants who lived further than 10 miles away reporting AC. In males, no other employment characteristics were associated with AC. In females, there was a suggestion that women whose job involved walking were more likely to use AC (adjust OR: 1.20 95%CI 1.00, 1.43).

Model 3 explored possible environmental correlates of AC behaviour. The only variable associated with AC was population density, in males only. The males who lived in a rural area had a 78% reduced odds of AC compared to those who lived in an urban or fringe area (adjusted OR 0.12 95% CI 0.02, 0.90). No other environmental variables were associated with AC.

DISCUSSION

A substantial majority (80%) of adults with T2DM reported commuting by car. AC was reported by 5.5% of the sample, of whom less than 1% reported cycling. AC was strongly and consistently associated with higher PA, whether expressed as MVPA or total MET-minutes/week. In fully adjusted models, AC was associated with an additional 73 minutes/week of MVPA for males and 105 minutes/week of MVPA for females, making a substantial contribution to meeting the UK Government recommended levels of 150 minutes/week MVPA [7]. This finding supports previous work by Audrey et al [19] which found levels of PA to be higher in those who walked to work compared to those who did not. Furthermore the association with MVPA seen in the present study is substantially larger than achieved in a similar population undergoing an intensive PA intervention [8], suggesting that adoption of AC could be an effective intervention for people with T2D.

The importance of sedentary time for both prevention and management of type 2 diabetes has gained research prominence in recent years [31,32], and we found a substantial association between AC and lower sedentary time. In both sexes both AC and public and mixed modes of transport were associated with over an hour less sedentary time per day compared to car use. Since adults with T2DM typically spend less than 3% of the day engaged in MVPA, interventions which focus on reducing sedentary time may be more effective to improve health [33]; re-allocating 30 minutes of prolonged sedentary time to more active pursuits can improve body composition and cholesterol levels in adults with T2DM [34].

The predominant correlate of AC was distance to work with few people who lived over 1.5 miles from their workplace choosing AC and those living in rural areas less likely to actively commute. Although distance from home to work is not modifiable, AC as the whole or part of

For peer review only - http://bmjopen.bmj.com/site/about/guidelines.xhtml

the journey could be encouraged by providing more cycleways, by road safety measures to limit traffic speed and improving access to parks and green spaces [35][36]. We were unable to examine the association of access to transport as a correlate of active commuting but it can be hypothesised this would correlate strongly with the transport method chosen.

. In the current study, the results for public transport were mixed. This may be due to an inability to differentiate between the different types of public transport taken and the public transport category also including people who reported mixed modes of motorised travel and active commuting. There are likely to be variations in the energy requirements of different transport modes which should be examined. It could also be hypothesised that walking or activity behaviours associated with public transport are difficult to recall and record in the IPAQ. However, in previous research public transport has been shown to contribute to PA and therefore warrants further investigation.

This study has some major strengths. UK Biobank provides a large, geographically diverse data source allowing multiple social, environmental and employment related factors to be adjusted for in analyses. The study sample is restricted to adults with T2DM and is the first study of its kind to explore these behaviours on such a scale.

The study also has some important limitations. The analyses were cross-sectional and therefore causality cannot be determined. The small numbers of participants reporting AC did not allow walking and cycling to be examined separately. The relative energy demands of these behaviours differ and previous research has suggested these behaviours are performed by different portions of society [37]. The current study was also unable to distinguish between the different modes of public transport. The observed gender difference suggests there may be differences in the work and travel patterns of males and females in this study population. The average distance from home to work was almost twice as far for males which may explain why more females reported active modes of travel. The study is further limited by self-report measures of PA and sedentary behaviour.

The study sample is predominantly of White British ethnicity which does not reflect the distribution of type 2 diabetes in the UK limiting the generalisability of the findings. The sample is also limited to those who work and therefore can only examine the role of active commuting on PA and sedentary time. The contribution of non-work active travel may be of more interest for people with T2DM who in general are older than the UK average.

In conclusion, AC is associated with increased PA, and reduced sedentary time in people with type 2 diabetes. This may have a beneficial effect on glucose control and could contribute towards reduced treatment costs. Strategies to increase active commuting in

For peer review only - http://bmjopen.bmj.com/site/about/guidelines.xhtml

adults with T2DM may increase levels of physical activity, and thus contribute to reducing the burden of this disease.

FUNDING

This project was funded by a small project grant from the National Institute for Health Research Bristol Nutrition Biomedical Research Unit. The funding covered the cost of data access only.

AUTHORS CONTRIBUTIONS

CF, AC and EF made substantial contributions to the conception and design, acquisition of data, or analysis and interpretation of data. All authors drafted the article or revised it critically for important intellectual content. All authors approved the final version of the manuscript to be published.

DATA SHARING STATEMENT

No additional data is available.

COMPETING INTEREST STATEMENT

There are no competing interests to declare by any authors.

REFERENCES

1 Hu FB, Manson JE, Stampfer MJ, *et al.* Diet, lifestyle, and the risk of type 2 diabetes mellitus in women. *N Engl J Med* 2001;**345**:790–7. doi:10.1056/NEJMoa010492

2 Kumari M, Head J, Marmot M, *et al.* Prospective Study of Social and Other Risk Factors for Incidence of Type 2 Diabetes in the Whitehall II Study. *Arch Intern Med* 2004;**164**:1873. doi:10.1001/archinte.164.17.1873

3 WHO (World Health Organisation). Global Report on Diabetes. *WHO* Published Online First: 2016.http://apps.who.int/iris/bitstream/10665/204871/1/9789241565257_eng.pdf?ua=1 (accessed 22 Jul2016).

4 Hex N, Bartlett C, Wright D, *et al.* Estimating the current and future costs of Type 1 and Type 2 diabetes in the UK, including direct health costs and indirect societal and productivity costs. *Diabet Med* 2012;**29**:855–62. doi:10.1111/j.1464-5491.2012.03698.x

5 Mary G, Caroline H, Naomi H, *et al.* Adult obesity and type 2 diabetes. Published Online First: 2014.www.gov.uk/phe (accessed 6 Jul2016).

6 Sigal RJ, Kenny GP, Wasserman DH, *et al.* Physical activity/exercise and type 2 diabetes: a consensus statement from the American Diabetes Association. *Diabetes Care* 2006;**29**:1433–8. doi:10.2337/dc06-9910

7 Chief Medical Officer. Physical activity guidelines for Adults (19-64 years). 2011.

8 Andrews RC, Cooper AR, Montgomery AA, *et al.* Diet or diet plus physical activity

For peer review only - http://bmjopen.bmj.com/site/about/guidelines.xhtml

versus usual care in patients with newly diagnosed type 2 diabetes: the Early ACTID randomised controlled trial. *Lancet (London, England)* 2011;**378**:129–39. doi:10.1016/S0140-6736(11)60442-X

9 JAKICIC JM, GREGG E, KNOWLER W, *et al.* Activity Patterns of Obese Adults with Type 2 Diabetes in the Look AHEAD Study. *Med Sci Sport Exerc* 2010;**42**:1995–2005. doi:10.1249/MSS.0b013e3181e054f0

10 Grøntved A, Hu FB. Television viewing and risk of type 2 diabetes, cardiovascular disease, and all-cause mortality: a meta-analysis. *JAMA* 2011;**305**:2448–55. doi:10.1001/jama.2011.812

11 Cassidy S, Chau JY, Catt M, *et al.* Cross-sectional study of diet, physical activity, television viewing and sleep duration in 233,110 adults from the UK Biobank; the behavioural phenotype of cardiovascular disease and type 2 diabetes. *BMJ Open* 2016;**6**:e010038. doi:10.1136/bmjopen-2015-010038

12 Physical activity: walking and cycling | Guidance and guidelines | NICE.

13 FPH. Take action on active travel. Fac. Public Heal. 2008.http://www.fph.org.uk/uploads/Take_action_on_active_travel.pdf (accessed 15 May2016).

14 Laverty research fellow AA, Hérick de Sá student T, Monteiro professor CA. Getting sedentary people moving through active travel. *J Epidemiol Community Heal* 2012;**66**:176–80. doi:10.1136/bmj.h725

15 Goodman A, Hamer M, Chida Y, *et al.* Walking, Cycling and Driving to Work in the English and Welsh 2011 Census: Trends, Socio-Economic Patterning and Relevance to Travel Behaviour in General. *PLoS One* 2013;**8**:e71790. doi:10.1371/journal.pone.0071790

16 Flint E, Cummins S, Sacker A. Associations between active commuting, body fat, and body mass index: population based, cross sectional study in the United Kingdom. BMJ (Clinical Res. ed). 2014. doi:10.1136/bmj.g4887 <http://dx.doi.org/10.1136/bmj.g4887>

17 Flint E, Cummins S. Does active commuting protect against obesity in mid-life? Cross-sectional, observational evidence from UK Biobank. *Lancet* Published Online First: 2015. doi:10.1016/S0140-6736(15)00846-6

18 Laverty AA, Mindell JS, Webb EA, *et al.* Active Travel to Work and Cardiovascular Risk Factors in the United Kingdom. *Am J Prev Med* 2013;**45**:282–8. doi:10.1016/j.amepre.2013.04.012

19 Audrey S, Procter S, Cooper AR, *et al.* The contribution of walking to work to adult physical activity levels: a cross sectional study. *Int J Behav Nutr Phys Act* 2014;**11**:37. doi:10.1186/1479-5868-11-37

20 Flint E, Webb E, Cummins S. Change in commute mode and body-mass index: prospective, longitudinal evidence from UK Biobank. *Lancet Public Heal* 2016;**1**:e46–55. doi:10.1016/S2468-2667(16)30006-8

21 Stewart G, Anokye NK, Pokhrel S. What interventions increase commuter cycling? A systematic review. *BMJ Open* 2015;**5**:e007945. doi:10.1136/bmjopen-2015-007945

22 Frank LD, Saelens BE, Powell KE, *et al.* Stepping towards causation: Do built environments or neighborhood and travel preferences explain physical activity, driving, and obesity? *Soc Sci Med* 2007;**65**:1898–914. doi:10.1016/j.socscimed.2007.05.053

For peer review only - http://bmjopen.bmj.com/site/about/guidelines.xhtml

23 Allen NE, Sudlow C, Peakman T, *et al.* UK biobank data: come and get it. *Sci Transl Med* 2014;**6**:224ed4. doi:10.1126/scitranslmed.3008601

24 Biobank U. Rationale, design and development of a large-scale prospective resource. UK Biobank. 2007.http://www.ukbiobank.ac.uk/resources/

25 Craig CL, Marshall AL, Sjöström M, *et al.* International physical activity questionnaire: 12-country reliability and validity. *Med Sci Sports Exerc* 2003;**35**:1381–95. doi:10.1249/01.MSS.0000078924.61453.FB

26 IPAQ. Guidelines for Data Processing and Analysis of the International Physical Activity Questionnaire (IPAQ) - Short Form,.

27 Small area health statistics unit. www.sashu.org

28 BioSHaRE EU Determinants of Health Project. http://bioshare.eu

29 Eeftens M, Beelen R, Hoogh K de, *et al.* Development of Land Use Regression Models for PM2.5, PM2.5 Absorbance, PM10 and PMcoarse in 20 European Study Areas; Results of the ESCAPE Project. 2012.

30 StataCorp 2015. Stata statistical software: Release 14.

31 Brocklebank LA, Falconer CL, Page AS, *et al.* Accelerometer-measured sedentary time and cardiometabolic biomarkers: A systematic review. *Prev Med (Baltim)* Published Online First: 2015. doi:10.1016/j.ypmed.2015.04.013

32 Owen N, Sparling PB, Healy GN, *et al.* Sedentary behavior: emerging evidence for a new health risk. *Mayo Clin Proc* 2010;**85**:1138–41. doi:10.4065/mcp.2010.0444

33 Falconer CL, Cooper AR, Walhin JP, *et al.* Sedentary time and markers of inflammation in people with newly diagnosed type 2 diabetes. *Nutr Metab Cardiovasc Dis* 2014;**24**:956–62. doi:10.1016/j.numecd.2014.03.009

34 Falconer CL, Page AS, Andrews RC, *et al.* The Potential Impact of Displacing Sedentary Time in Adults with Type 2 Diabetes. *Med Sci Sports Exerc* 2015;**47**:2070–5. doi:10.1249/MSS.0000000000000651

35 Mackenbach JD, Rutter H, Compernolle S, *et al.* Obesogenic environments: a systematic review of the association between the physical environment and adult weight status, the SPOTLIGHT project. *BMC Public Health* 2014;**14**:233. doi:10.1186/1471-2458-14-233

36 Tomlinson P, Hewitt S, Blackshaw N. Joining up health and planning: how Joint Strategic Needs Assessment (JSNA) can inform health and wellbeing strategies and spatial planning. *Perspect Public Health* 2013;**133**:254–62. doi:10.1177/1757913913488331

37 Mytton OT, Panter J, Ogilvie D. Longitudinal associations of active commuting with wellbeing and sickness absence. *Prev Med (Baltim)* 2016;**84**:19–26. doi:10.1016/j.ypmed.2015.12.010

For peer review only - http://bmjopen.bmj.com/site/about/guidelines.xhtml

For peer review only

For peer review only - http://bmjopen.bmj.com/site/about/guidelines.xhtml

For peer review only

For peer review only - http://bmjopen.bmj.com/site/about/guidelines.xhtml

For peer review only

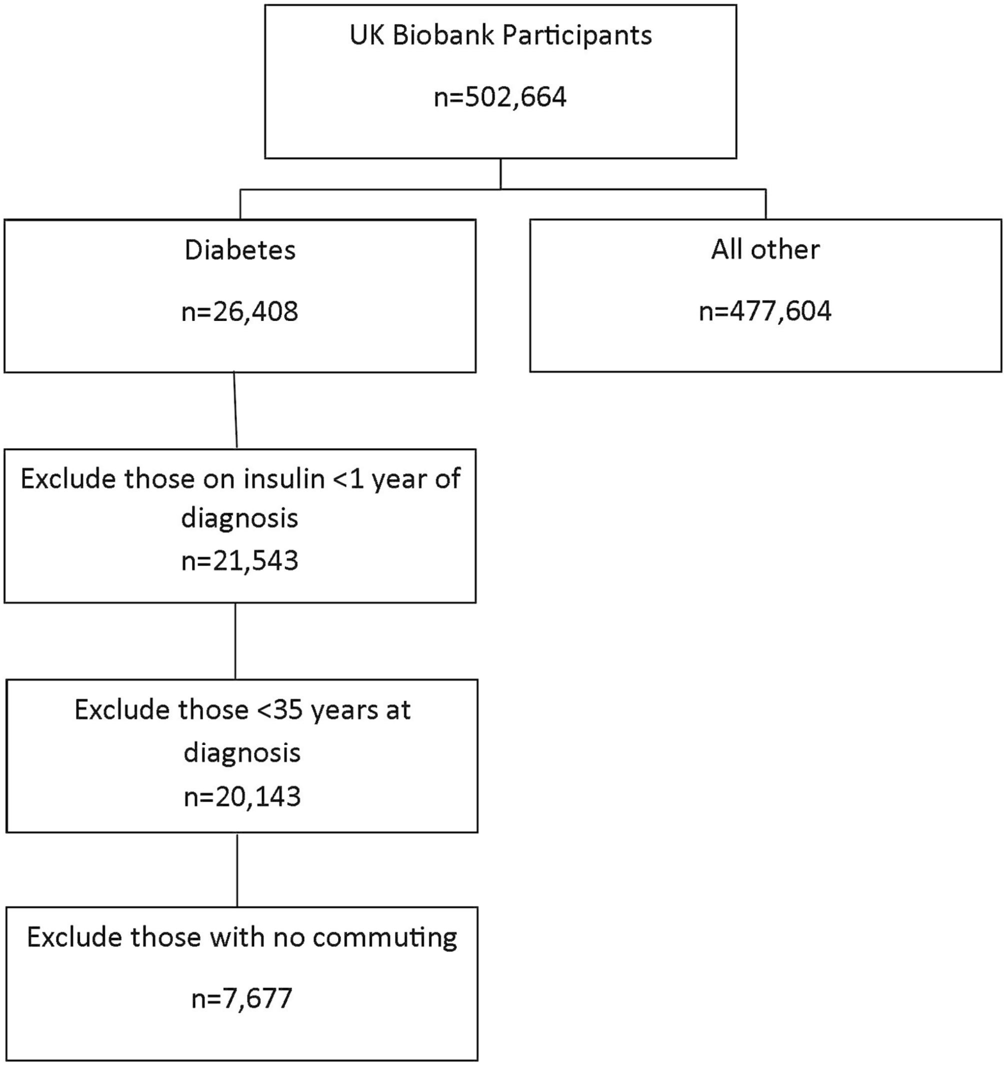

Figure 1. Flow chart demonstrating how population sample was identified.

135x142mm (300 x 300 DPI)

STROBE Statement—checklist of items that should be included in reports of observational studies

	Item No	Recommendation	Page number
**Title and abstract**	1	(*a*) Indicate the study's design with a commonly used term in the title or the abstract	1
		(*b*) Provide in the abstract an informative and balanced summary of what was done and what was found	2
**Introduction**			
Background/rationale	2	Explain the scientific background and rationale for the investigation being reported	4-5
Objectives	3	State specific objectives, including any prespecified hypotheses	5
**Methods**			
Study design	4	Present key elements of study design early in the paper	6
Setting	5	Describe the setting, locations, and relevant dates, including periods of recruitment, exposure, follow-up, and data collection	6
Participants	6	(*a*) *Cohort study*—Give the eligibility criteria, and the sources and methods of selection of participants. Describe methods of follow-up<br>*Case-control study*—Give the eligibility criteria, and the sources and methods of case ascertainment and control selection. Give the rationale for the choice of cases and controls<br>*Cross-sectional study*—Give the eligibility criteria, and the sources and methods of selection of participants	6
		(*b*) *Cohort study*—For matched studies, give matching criteria and number of exposed and unexposed<br>*Case-control study*—For matched studies, give matching criteria and the number of controls per case	
Variables	7	Clearly define all outcomes, exposures, predictors, potential confounders, and effect modifiers. Give diagnostic criteria, if applicable	6-7
Data sources/ measurement	8*	For each variable of interest, give sources of data and details of methods of assessment (measurement). Describe comparability of assessment methods if there is more than one group	6-7
Bias	9	Describe any efforts to address potential sources of bias	7
Study size	10	Explain how the study size was arrived at	NA
Quantitative variables	11	Explain how quantitative variables were handled in the analyses. If applicable, describe which groupings were chosen and why	8
Statistical methods	12	(*a*) Describe all statistical methods, including those used to control for confounding	8
		(*b*) Describe any methods used to examine subgroups and interactions	88
		(*c*) Explain how missing data were addressed	
		(*d*) *Cohort study*—If applicable, explain how loss to follow-up was addressed<br>*Case-control study*—If applicable, explain how matching of cases and controls was addressed<br>*Cross-sectional study*—If applicable, describe analytical methods taking account of sampling strategy	8
		(*e*) Describe any sensitivity analyses	8

Continued on next page

For peer review only

**Results**			
Participants	13*	(a) Report numbers of individuals at each stage of study—eg numbers potentially eligible, examined for eligibility, confirmed eligible, included in the study, completing follow-up, and analysed	
		(b) Give reasons for non-participation at each stage	
		(c) Consider use of a flow diagram	
Descriptive data	14*	(a) Give characteristics of study participants (eg demographic, clinical, social) and information on exposures and potential confounders	8
		(b) Indicate number of participants with missing data for each variable of interest	
		(c) *Cohort study*—Summarise follow-up time (eg, average and total amount)	
Outcome data	15*	*Cohort study*—Report numbers of outcome events or summary measures over time	8
		Case-control study—Report numbers in each exposure category, or summary measures of exposure	
		Cross-sectional study—Report numbers of outcome events or summary measures	
Main results	16	(*a*) Give unadjusted estimates and, if applicable, confounder-adjusted estimates and their precision (eg, 95% confidence interval). Make clear which confounders were adjusted for and why they were included	8-10
		(*b*) Report category boundaries when continuous variables were categorized	
		(*c*) If relevant, consider translating estimates of relative risk into absolute risk for a meaningful time period	
Other analyses	17	Report other analyses done—eg analyses of subgroups and interactions, and sensitivity analyses	8-10
**Discussion**			
Key results	18	Summarise key results with reference to study objectives	10-11
Limitations	19	Discuss limitations of the study, taking into account sources of potential bias or imprecision. Discuss both direction and magnitude of any potential bias	12
Interpretation	20	Give a cautious overall interpretation of results considering objectives, limitations, multiplicity of analyses, results from similar studies, and other relevant evidence	12
Generalisability	21	Discuss the generalisability (external validity) of the study results	12
**Other information**			
Funding	22	Give the source of funding and the role of the funders for the present study and, if applicable, for the original study on which the present article is based	13

*Give information separately for cases and controls in case-control studies and, if applicable, for exposed and unexposed groups in cohort and cross-sectional studies.

Note: An Explanation and Elaboration article discusses each checklist item and gives methodological background and published examples of transparent reporting. The STROBE checklist is best used in conjunction with this article (freely available on the Web sites of PLoS Medicine at http://www.plosmedicine.org/, Annals of Internal Medicine at http://www.annals.org/, and Epidemiology at http://www.epidem.com/). Information on the STROBE Initiative is available at www.strobe-statement.org.

For peer review only

For peer review only - http://bmjopen.bmj.com/site/about/guidelines.xhtml

BMJ Open

Patterns and correlates of active commuting in adults with type 2 diabetes: cross-sectional evidence from UK Biobank

Journal:	*BMJ Open*
Manuscript ID	bmjopen-2017-017132.R2
Article Type:	Research
Date Submitted by the Author:	13-Jul-2017
Complete List of Authors:	Falconer, Catherine; Gloucestershire Hospitals NHS Foundation Trust, ; University of Bristol, NIHR Bristol Nutrition Biomedical Research Unit<br>Cooper, Ashley; University of Bristol<br>Flint, Ellen; London School of Hygiene and Tropical Medicine, Social and Environmental Health Research
**Primary Subject Heading**:	Public health
Secondary Subject Heading:	Sports and exercise medicine
Keywords:	General diabetes < DIABETES & ENDOCRINOLOGY, EPIDEMIOLOGY, PREVENTIVE MEDICINE, PUBLIC HEALTH

SCHOLARONE™
Manuscripts

Title Page

Patterns and correlates of active commuting in adults with type 2 diabetes: cross-sectional evidence from UK Biobank

Falconer, Catherine L[1,2]; Cooper, Ashley R[2,3]; Flint, Ellen[4]

[1]NHS Gloucestershire Foundation Trust,

[2]NIHR Bristol Nutrition Biomedical Research Unit, University of Bristol

[3]Centre for Exercise, Nutrition and Health Sciences, University of Bristol

[4] Department of Social and Environmental Health Research, London School of Hygiene and Tropical Medicine

For peer review only

Word Count: 3,269
Abstract: 280
Number of tables: 3
Number of figures: 0

Corresponding author:

Professor Ashley Cooper
Bristol Nutrition BRU
Level 3, University Hospitals Research and Education Centre,
Upper Maudlin Street,
Bristol,
BS2 8AE
Email: Ashley.cooper@bristol.ac.uk

ABSTRACT

Objectives To describe the active commuting patterns of adults with type 2 diabetes and how these relate to physical activity and sedentary behaviour in UK Biobank. Social and environmental correlates of active commuting will also be explored.

Design Cross-sectional analysis of a cohort study

Settings Population cohort of over 500,000 people recruited from 22 centres across the United Kingdom. Participants aged between 37 and 73 years were recruited between 2006 and 2010.

Participants 6,896 participants with a self-reported type 2 diabetes diagnosis who reported commuting to work and had complete covariate data were included in the analysis.

Exposure measures Exposure measures were active commuting (AC) to work, measured as usual mode of transport.

Outcome measures Outcome measures were weekly minutes of moderate-to-vigorous physical activity (MVPA), and hours/day of sedentary time, and participation in active travel.

Results AC (reporting walking or cycling to work only) was reported by 5.5% of participants with the overwhelming majority using the car to commute (80%). AC was associated with an additional 73 (95%CI 10.8, 134.9) and 105 (95%CI 41.7, 167.2) weekly minutes of MVPA for males and females respectively. AC was associated with reduced sedentary time (β-1.1 95%CI -1.6, -0.7 hours/day for males and β-0.8 95%CI -1.2, -0.3 hours/day for females). Deprivation and distance from home to work were identified as correlates of AC behaviour.

Conclusions Rates of active commuting are very low in adults with type 2 diabetes. However, active commuting offers a potentially sustainable solution to increasing physical activity and reducing sedentary behaviour. Therefore, strategies to improve the environment and encourage active commuting may help to increase population levels of physical activity and reduce the disease burden associated with type 2 diabetes.

For peer review only - http://bmjopen.bmj.com/site/about/guidelines.xhtml

Article summary

Strengths and limitations of this study

This study uses the UK Biobank to examine the active commuting behaviours of adults with type 2 diabetes. UK Biobank is a large, geographically diverse cohort which provides the largest exploration of active commuting in this population to date.

UK Biobank provided the opportunity to be able to explore a multitude of employment, social and environmental factors which may contribute to active commuting.

We were unable to distinguish between walking and cycling behaviour, and also were unable to explore different type of public transport modes for example bus or train.

The analysis used in this study is cross-sectional and therefore we cannot infer causality.

This study is further limited by the use of self-report measures of physical activity and sedentary time.

For peer review only

For peer review only - http://bmjopen.bmj.com/site/about/guidelines.xhtml

INTRODUCTION

Type 2 diabetes mellitus (T2DM) is a disease associated with unhealthy lifestyle behaviours, such as poor diet and physical inactivity [1] and displays a social gradient [2]. The worldwide age-standardised prevalence of T2DM has doubled to 8.5% since 1980 [3]. In the UK the prevalence is 6% and the ongoing treatment and management burden of T2DM and its associated complications places a considerable burden on the National Health Service [4,5].

The role of physical activity (PA) in the prevention and management of T2DM is well understood [6]. However, fewer adults with T2DM achieve UK Government recommendations of at least 150 minutes moderate-to-vigorous physical activity (MVPA) per week [7] and many interventions fail to achieve increases in PA sufficient to confer metabolic benefits [7–9]. Sedentary behaviour is also an independent risk factor for the development of T2DM [10]. Cross-sectional evidence from UK Biobank suggests that adults with T2DM are characterised by a phenotype of unhealthy behaviours including prolonged sedentary time, physical inactivity and poor sleep, suggesting that these behaviours should be targeted in parallel [11].

The role of active commuting in increasing PA and controlling excessive weight gain has been recognised and endorsed by the National Institute for Health and Care Excellence (NICE) [12]. Active commuting has the potential to increase individuals PA and reduce sedentary time, and more widely to lower levels of traffic congestion and noise and air pollution [13], and can be sustainably incorporated into daily living [14]. However, rates of AC are low in the UK. In the 2011 census, of the 58% of adults who responded to the question ‘how do you usually travel to work?, 67% reported private motorised transport as the principle mode of transport,17.8% public transport, 10.9% walking and 3.1% commuting by bicycle [15].

There is increasing evidence of an association between AC and measures of obesity [16,17]. In the UK Understanding Society cohort, active commuting has been shown to be predictive of lower BMI and body fat percentage, and a lower adjusted odds of reporting a diabetes diagnosis compared to car users [16][18]. Similarly in UK Biobank, AC was significantly and independently associated with lower BMI and body fat percentage [17]. The protective effect of AC is likely to be mediated through increased PA as demonstrated in a UK study which used accelerometry and GPS receivers to show that walking to work contributed 47.3% of daily MVPA [19].

Causal evidence from longitudinal or experimental studies is rare. A longitudinal analysis of the UK Biobank cohort looked at changes in BMI in response to changes in commuting mode over a 4 year follow up. Individuals who transitioned from car commuting at baseline to

For peer review only - http://bmjopen.bmj.com/site/about/guidelines.xhtml

active or public transportation modes at follow up had a decrease in BMI of -0.30kg/m^2 (95%CI -0.47, -0.13) [20].

Promoting AC may help to increase population levels of activity; however, there is a need to understand the correlates of AC to develop appropriate interventions. Some key determinants of commuter cycling and walking behaviour have been identified as high population density, dedicated cycling paths, short trip distances and good traffic and road conditions, such as the presence of traffic calming measures [21][22]. Most work to date has been performed in healthy adults and there is a need to understand how these findings relate to populations with T2DM who are characterised by obesity and physical inactivity.

The primary aim of this work is to describe the AC patterns of adults with T2DM in UK Biobank. Specifically, the study explores how AC is associated with PA and sedentary time in this population (objective 1). The study will also aim to identify social and environmental factors (age, social class and traffic density) that may predict participation in AC (objective 2).

METHODS

Study population

Baseline data from the UK Biobank study were used (project 19307). UK Biobank is a large population study which aims to examine how the environment, lifestyle and genes interact to affect health [23]. Full recruitment procedures are described in detail elsewhere, [24] and briefly summarised below.

INSERT FIGURE 1Between 2006 and 2010, recruitment invitations were sent to 9.2 million people from 22 assessment centres around the UK to obtain a sample of 502,565 adults aged 37-73 years [11]. Following recruitment, participants provided written, informed consent and attended an assessment centre for data collection. A touchscreen questionnaire covered socio-demographics, occupation, lifestyle, early exposures, cognitive function, family history and medical history. Participants also underwent a verbal interview, physical measures and provided a blood/urine sample. All data were anonymised. The UK Biobank Access Subcommittee granted approval for the data access under a Material Transfer Agreement with the London School of Hygiene and Tropical Medicine who provided ethical approval for this project (LSHTM Ethics reference: 10838).

Study sample

Participants who reported having diabetes or T2DM were selected (n=26,408). We excluded anyone <35 years at diagnosis of diabetes and who reported taking insulin within the first

For peer review only - http://bmjopen.bmj.com/site/about/guidelines.xhtml

year of diagnosis to reduce the likelihood of type 1 or monogenic forms of diabetes [11], (n=20,143). Only participants who reported commuting behaviours and had complete covariate data were included in the analysis (n=6,896).

Active commuting measures

In response to the question "what types of transport do you use to get to and from work?", participants could select one or more of the following options: car/motor vehicle; walk; public transport; cycle. In order to answer the two study objectives, two distinct exposure variables were derived: 1. a three-category exposure variable; (1) *car* or *car and public transport*; (2) *public transport* and *mixed* (car and walk/cycle; public transport and walk/cycle); (3) AC: *walk* or *cycle* only and 2. a binary variable ((1) No active commuting; (2) Active commuting (AC: walk or cycle only)). Only participants who exclusively reported walking or cycling behaviour were included in the active commuting categories.

Physical activity

PA was assessed using questions adapted from the validated short form of the International Physical Activity Questionnaire (IPAQ) [25]. The questions asked participants to self-report the frequency, duration and intensity of walking, moderate and vigorous activity. From these questions, we derived three measures of physical activity. A variable for total physical activity (total MET-minutes/week) was derived by weighting time spent in vigorous, moderate and walking activity by the relevant energy expended for each activity to produce metabolic equivalents, MET-minutes per week of PA [26]. Duration of MVPA was derived as the sum of reported duration and frequency of moderate and vigorous activity. From this, a variable for 'attainment of physical activity guidelines' (150 minutes' moderate PA or 75 minutes of vigorous activity per week) was derived[7].

Sedentary behaviour

Self-reported television viewing, computer use and driving behaviour were collected from the touchscreen questionnaire. An estimate of average total sedentary time was derived by summing the daily reported duration of television viewing, computer use and driving. Total sedentary time is expressed as hours per day.

Environmental correlates

Local environmental exposure variables were collated by the Small Area Health Statistics Unit [27] as part of the BioSHaRE-EU Environmental Determinants of Health Project [28]. Air pollution estimates were modelled for the year 2010 using a Land Use Regression (LUR) model developed as part of the European Study of Cohorts for Air Pollution Effects (ESCAPE) [29]. ESCAPE estimates of particulate matter (PM) are valid up to 400km from

For peer review only - http://bmjopen.bmj.com/site/about/guidelines.xhtml

For peer review only

the monitoring area, which in this case was Greater London. Therefore, participants who have an address more than 400km from this site were not assigned a value for PM2.5-10. Traffic variables, including inverse distance to a major road and traffic density, were also calculated using the LUR model, based on Eurostreets (v3.1) digital road network for the year 2008. Traffic density is measured as the average total number of motor vehicles per 24 hours on the nearest road. These correlates were selected on the basis that they were available on an individual level and may help to inform further analysis of environmental correlates of commuting behaviour.

Covariates

A range of confounding variables were considered. Socioeconomic covariates were; Townsend Deprivation Index of residential area, annual gross household income, highest educational qualification. Further demographic covariates were: age (years); sex; ethnicity, census-derived residential area population density classification (urban/fringe, rural). The potential confounding effects of occupational physical activity were considered: job involves standing/walking; job involves heavy manual/physical work, shift work, non-work related transport modes (active modes: yes/no). Health covariates were: poor self-rated health (yes/no). Commute frequency (number of outward journeys per week, truncated at 10) and distance between home and workplace (reported in miles) were included as covariates in the model.

Statistical analyses

Descriptive analyses were undertaken to describe the commuting patterns of the population, the prevalence of PA and sedentary behaviours and the patterning of hypothesised confounding variables. Continuous variables are displayed as mean and standard deviation and frequencies are used for categorical variables.

To address objective 1, multivariate linear regression was used to describe the association between AC and each of the two outcome variables (PA and sedentary time) adjusting for potential confounding variables in two step-wise series of three nested models. The first model presents the unadjusted association between the exposure (commuting mode) and outcome of interest. The second model adjusts for demographic and socio-economic factors. The third model additionally adjusts for employment-related confounders such as distance from home to work.

To address objective 2, multivariate logistic regression modelling was used to explore potential socio-demographic, environmental and employment-related correlates of AC behaviour. The model used was adjusted for all included variables. All analyses controlled for geographical clustering by assessment centre, and potential interactions by gender,

For peer review only - http://bmjopen.bmj.com/site/about/guidelines.xhtml

ethnicity and household income were explored. For these analyses, the outcome was expressed as a binary categorical variable ((1) not active commuting, (2) active commuting). All analyses were conducted using Stata v14 [30].

RESULTS

The final sample was 6,896 participants with T2DM who reported commuting behaviour and provided complete data on all covariates (Table I).

Table 1. Demographic, commuting behaviour, employment, physical activity and environmental characteristics of the study population

Variable	Males (n=4,473)	Females (n=2,423)	Total (n=6,896)
**Demographics**			
Age (years)	56.7 ± 6.5	54.53 ± 6.6	55.9 ± 6.6
Ethnicity (%)			
White British	81.8	78.2	80.5
Other white	5.3	6.5	5.7
South Asian	6.2	4.2	5.5
Black Caribbean	1.3	3.9	2.2
Black African	1.8	2.1	1.9
Chinese	0.3	0.6	0.4
Mixed ethnicity	0.6	0.8	0.7
Other ethnicity	2.7	3.8	3.1
Townsend Index of deprivation(quintiles) (%)			
1(most deprived)	16.1	14.1	15.4
2	17.2	15.8	16.7
3	19.6	20.9	20.1
4	21.2	22.1	21.8
5 (least deprived)	25.5	27.1	26.0
Highest Educational Qualification (%)			
University	29.6	30.8	30.0
A Levels/AS Levels	11.2	11.2	11.4
GCSE/O Levels	20.5	25.0	22.1
CSE's or equivalent	6.4	7.9	6.9
NVQ or HND or HNC	11.5	7.0	9.9
Other professional qualifications	5.32	6.2	5.6
None	15.6	11.6	14.2
Average household income (%)			
<£18,000	12.2	20.5	15.1
£18,000 to £30,999	27.4	28.0	27.6
£31,000 to £51,999	30.9	29.3	30.4
£52,000 to £100,000	23.8	18.6	22.0
>£100,000	5.7	3.5	4.9
Poor self-rated health (%)	9.9	9.0	9.6
**Employment**			
Job involves heavy lifting (%)			
Never/rarely	59.8	65.8	61.9
Sometimes	25.0	22.5	24.1
Usually	8.1	5.7	7.2
Always	7.1	6.0	6.7
Job involves walking(%)			
Never/rarely	31.3	32.8	31.8
Sometimes	32.7	31.2	32.2
Usually	16.1	13.8	15.3
Always	19.9	22.2	20.7
Shift work	7.7	5.9	7.1
**Commuting behaviour**			

For peer review only - http://bmjopen.bmj.com/site/about/guidelines.xhtml

For peer review only

Commute frequency (no. outward journeys/week)	4.6 ± 2.1	4.3 ± 1.8	4.5 ± 2.0
Distance from home to work (miles)	14.9 ± 28.4	7.5 ± 9.9	12.3 ± 24.0
Commuting mode (%)			
Motorised travel	83.2	75.3	80.4
Motorised travel plus active travel	12.3	17.5	14.1
Active commuting	4.5	7.2	5.4
Non work active travel	40.4	40.2	40.3
**Physical activity and sedentary time**			
Daily sedentary time (hours)	6.0 ± 3.1	4.9 ± 2.7	5.6 ± 3.0
Total weekly MVPA[1] (minutes)	263.9 ± 446.4	214.9 ± 383.4	246.6 ± 425.9
Total weekly MET[2]-minutes	2373.3 ± 3099.8	2014.7 ± 2854.7	2245.5 ± 3106.2
Achieves PA guidelines[3] (%)	54.3	53.8	54.1
**Environmental characteristics**			
Close to major road (%)	8.0	8.2	8.1
Population density (urban/fringe) (%)	94.5	95.7	94.9
Inverse distance to nearest road (1/m)	0.05 (0.05, 0.05)	0.05 (0.05, 0.05)	0.05 (0.05, 0.05)
Air pollution PM2.5-10 (micro-g/m^3)	6.5 ± 0.9	6.5 ± 0.9	6.5 ± 0.9
Traffic density (vehicles/day)	1699.4 ± 5663.6	1716.2 ± 6057.0	1698.6 ± 5663.6

[1]MVPA, Moderate to vigorous physical activity; [2]MET, metabolic equivalents (1MET=1kcal/kg/hr); [3]PA guidelines, physical activity guidelines (150 minutes MVPA/week or 75 minutes vigorous activity/week)[7]

Car travel was reported by 83.2% of male and 75.2% of female participants, with walking or cycling exclusively reported by 4.5% of males and 7.2% of females. Females spent less time sedentary than males (4.9 ± 2.7 hours/day versus 6.0 ± 3.1 hours/day), while males reported more weekly MVPA (263.9 ± 446.4 minutes/week versus 214.9 ± 383.4 minutes/week).

The results of analyses to investigate the association between commuting mode, PA and sedentary behaviour (objective 1) are displayed in table 2. A significant interaction for gender was found so stratified analyses are presented (p<0.1).

For peer review only

For peer review only - http://bmjopen.bmj.com/site/about/guidelines.xhtml

Table 2. Results of linear regression modelling to investigate the associations between commuting behaviour, physical activity and sedentary time, stratified by gender.

	MVPA (minutes/week)[1]		Total MET-minutes/week		Total sedentary time (hours/day)	
	Males β (95% CI)	Females β (95% CI)	Males β (95% CI)	Females β (95% CI)	Males β (95% CI)	Females β (95% CI)
**Model 1**						
Car (reference)	0.0	0.0	0.0	0.0	0.0	0.0
MT/AC[2]	-49.9 (-89.8, -10.1)	12.5 (-27.9, 52.8)	93.0 (-381.0, 193.0)	312.3 (12.3, 612.4)	-2.0 (-2.3, -1.7)	-1.0 (-1.3, 0.7)
Active commuting	86.4 (23.1, 149.7)	156.2 (97.0, 215.4)	777.0 (320.6, 1233.3)	1287.3 (847.3, 1727.4)	-1.3 (-1.8, -0.9)	-1.0 (-1.4, -0.6)
**Model 2[3]**						
Car (reference)	0.0	0.0	0.0	0.0	0.0	0.0
MT/AC[2]	-57.4 (-98.6, -16.2)	-3.0 (-45.9, 40.1)	-156.1 (-452.4, 140.3)	155.2 (-163.4, 473.9)	-2.2 (-2.4, -1.9)	-1.2 (-1.5, -0.9)
Active commuting	74.6 (10.9, 138.4)	145.3 (85.5, 205.2)	665.5 (207.0, 1124.1)	1149.4 (706.3, 1592.5)	-1.5 (-1.9, -1.0)	-1.1 (-1.5, -0.7)
**Model 3[4]**						
Car (reference)	0.0	0.0	0.0	0.0	0.0	0.0
MT/AC[2]	-29.6 (-70.6, 11.4)	11.5 (-35.3, 58.3)	47.0 (-240.9, 334.9)	290.8 (-46.0, 627.6)	-2.2 (-2.5, -1.9)	-1.1 (-1.5, -0.8)
Active commuting	72.9 (10.8, 134.9)	104.5 (41.7, 167.2)	586.6 (150.9, 1022.3)	722.3 (270.3, 1174.1)	-1.1 (-1.6, -0.7)	-0.8 (-1.2, -0.3)

[1]MVPA: Moderate to vigorous physical activity; [2]MT/AC: motorised travel and active commuting; [3]Model is adjusted for age, ethnicity, average household income, Townsend deprivation score, assessment centre, poor health; [4]additionally adjusted for distance from home to work, commute frequency, population density, non-work active travel and job involves walking.Model 1 present unadjusted associations.

For peer review only - http://bmjopen.bmj.com/site/about/guidelines.xhtml

In all models, AC was associated with higher MVPA. In fully adjusted models, compared to motorised travel, AC was associated with an additional 72.9 (95%CI 10.8, 134.9) minutes/week of MVPA for males and 104.5 (95%CI 41.7, 167.2) minutes/week for females. The association with motorised travel and active commuting was less clear. In males, there was a suggestion that motorised travel and active commuting were associated with less MVPA compared to motorised travel alone. This association was attenuated once environmental factors were included in the model (adjusted β 29.6; 95%CI -70.6, 11.4).

AC was also consistently associated with increased total PA in both genders, an effect which survived adjustment for all confounding factors (β 586.6; 95%CI 150.9, 1022.3 for males and β 722.3; 95%CI 270.3, 1174.1 for females). In unadjusted models, motorised travel and active commuting were associated with increased total PA for females only; however, this was attenuated once employment and demographic factors were included in the model.

In both males and females, and in all models, consistent associations were seen between commuting mode and total sedentary time. In fully adjusted models, compared to male motorised travellers, AC was associated with 1.1 (95%CI -1.6, -0.7) fewer hours/day sedentary while mixed motorised travel and active commuting modes were associated with 2.2 (95%CI 2.5, 1.9) fewer sedentary hours/day. A similar effect was seen in females with AC associated with 0.8 (95%CI -1.2, -0.3) fewer sedentary hours/day and mixed motorised travel and active commuting were associated with 1.1 (95%CI 1.5, 0.8) fewer sedentary hours/day compared to car users.

The results of multivariate logistic regression performed to identify social, employment and environmental correlates of AC behaviour (objective 2) are displayed in Table 3. There was evidence of a significant interaction for gender so stratified results are presented.

Table 3. Results of logistic regression modelling to identify potential social, employment and environmental correlates of active commuting.

	**Model 1 Individual factors**		**Model 2 Employment factors**		**Model 3 Environmental factors**	
	**Males OR (95% CI)**	**Females OR (95% CI)**	**Males OR (95% CI)**	**Females OR (95% CI)**	**Males OR (95% CI)**	**Females OR (95% CI)**
**Social factors**						
Age (years)	1.0 (0.9, 1.0)	0.98 (0.95, 1.00)	0.98 (0.96, 1.00)	0.97 (0.94, 0.99)	1.01 (0.98, 1.03)	0.97 (0.95, 0.99)
Townsend Deprivation quintile	1.0	1.0	1.0	1.0	1.0	1.0
1 (ref) 2	1.13 (0.58, 2.22)	2.09 (0.93, 4.71)	0.88 (0.42, 1.84)	2.76 (1.10, 6.92)	1.19 (0.60, 2.37)	1.99 (0.88, 4.50)
3 4	1.90 (1.03, 3.49)	3.00 (1.42, 6.37)	1.35 (0.69, 2.64)	2.54 (1.10, 5.90)	1.80 (0.96, 3.37)	2.69 (1.26, 5.75)
5	2.04 (1.12, 3.71)	3.25 (1.55, 6.83)	1.49 (0.77, 2.88)	2.60 (1.14, 5.95)	1.79 (0.96, 3.34)	2.92 (1.38, 6.19)
	4.24 (2.41,	3.34 (1.61,	2.28 (1.23,	3.07 (1.36,	3.32 (1.84,	3.08 (1.47,

For peer review only - http://bmjopen.bmj.com/site/about/guidelines.xhtml

	7.44)	6.94)	4.21)	6.94)	6.01)	6.48)
Average household income (£)	1.0	1.0	1.0	1.0	1.0	1.0
<£18,000 (ref)						
£18,000 to £30,999	0.68 (0.44, 1.04)	0.72 (0.48, 1.09)	0.97 (0.59, 1.59)	1.25 (0.77, 2.04)	0.61 (0.39, 0.97)	0.76 (0.50, 1.16)
£31,000 to £51,999						
£52,000 to £100,000	0.69 (0.44, 1.07)	0.39 (0.24, 0.62)	1.23 (0.74, 2.04)	0.94 (0.53, 1.65)	0.65 (0.42, 1.03)	0.41 (0.25, 0.67)
>£100,000	0.85 (0.53, 1.37)	0.35 (0.20, 0.62)	1.34 (0.77, 2.33)	0.88 (0.45, 1.74)	0.80 (0.49, 1.32)	0.37 (0.21, 0.67)
	1.10 (0.58, 2.11)	0.90 (0.40, 2.01)	1.27 (0.59, 2.79)	4.27 (1.47, 12.40)	0.90 (0.43, 1.89)	0.75 (0.30, 1.87)
Assessment centre	0.97 (0.95, 1.00)	1.00 (0.98, 1.03)	0.98 (0.95, 1.01)	1.02 (0.98, 1.05)	0.98 (0.95, 1.00)	1.00 (0.97, 1.03)
Poor health	0.70 (0.41, 1.18)	0.83 (0.47, 1.46)	0.56 (0.31, 1.00)	1.04 (0.53, 2.04)	0.62 (0.35, 1.11)	0.89 (0.50, 1.56)
**Employment characteristics**						
Distance from home to work (miles)						
<1.5 (ref)			1.0	1.0		
			0.12 (0.08, 0.17)	0.07 (0.05, 0.11)		
1.5-4						
			0.01 (0.01, 0.03)	0.01 (0.05, 0.03)		
4-10						
>10			0.002 (0.001, 0.01)	0.00		
Commute frequency			0.95 (0.86, 1.05)	1.01 (0.91, 1.14)		
Job involves walking			0.97 (0.83, 1.13)	1.20 (1.00, 1.43)		
Shift work			0.85 (0.43, 1.67)	0.76 (0.31, 1.88)		
**Environmental characteristics**						
Population density (ref: urban)					0.12 (0.02, 0.90)	0.39 (0.09, 1.62)
Air pollution (PM10 microg/m3)					1.01 (0.84, 1.21)	0.89 (0.72, 1.09)
Traffic density (no. cars)					1.0 (0.99, 1.0)	0.99 (0.99, 1.00)
Close to major road (ref: yes)					1.48 (0.76, 2.89)	1.41 (0.64, 3.09)
Distance to nearest road (1/miles)					0.15 (0.01, 4.15)	0.49 (0.02, 9.67)

In model 1 for both males and females, the odds of participating in AC increased with increasing neighbourhood deprivation score, with the strongest effect seen in deprivation quintile 5 compared to quintile 1 (adjusted OR:4.24 95%CI 2.41, 7.44 for males and adjusted OR:3.34 95%CI 1.61, 6.94 for females). In fully adjusted models no other social factors were associated with AC behaviour.

Model 2 additionally adjusted for employment-related factors. In females, neighbourhood deprivation remained a significant correlate of AC; however, for males the association was attenuated. Distance to work was the strongest correlate of AC. In males, the odds of AC were 88% lower (adjusted OR: 0.12 95%CI: 0.08, 0.17) in those who lived between 1.5 and 4 miles from work compared to those who lived within 1.5 miles. The odds of AC were 93%

For peer review only - http://bmjopen.bmj.com/site/about/guidelines.xhtml

lower (adjusted OR: 0.07 95%CI 0.05, 0.11) in females who lived between 1.5 and 4 miles from work with no participants who lived further than 10 miles away reporting AC. In males, no other employment characteristics were associated with AC. In females, there was a suggestion that women whose job involved walking were more likely to use AC (adjust OR: 1.20 95%CI 1.00, 1.43).

Model 3 explored possible environmental correlates of AC behaviour. The only variable associated with AC was population density, in males only. The males who lived in a rural area had a 78% reduced odds of AC compared to those who lived in an urban or fringe area (adjusted OR 0.12 95% CI 0.02, 0.90). No other environmental variables were associated with AC.

DISCUSSION

A substantial majority (80%) of adults with T2DM reported commuting by car. AC was reported by 5.5% of the sample, of whom less than 1% reported cycling. AC was strongly and consistently associated with higher PA, whether expressed as MVPA or total MET-minutes/week. In fully adjusted models, AC was associated with an additional 73 minutes/week of MVPA for males and 105 minutes/week of MVPA for females, making a substantial contribution to meeting the UK Government recommended levels of 150 minutes/week MVPA [7]. This finding supports previous work by Audrey et al [19] which found levels of PA to be higher in those who walked to work compared to those who did not. Furthermore the association with MVPA seen in the present study is substantially larger than achieved in a similar population undergoing an intensive PA intervention [8], suggesting that adoption of AC could be an effective intervention for people with T2D.

The importance of sedentary time for both prevention and management of type 2 diabetes has gained research prominence in recent years [31,32], and we found a substantial association between AC and lower sedentary time. In both sexes both AC and public and mixed modes of transport were associated with over an hour less sedentary time per day compared to car use. Since adults with T2DM typically spend less than 3% of the day engaged in MVPA, interventions which focus on reducing sedentary time may be more effective to improve health [33]; re-allocating 30 minutes of prolonged sedentary time to more active pursuits can improve body composition and cholesterol levels in adults with T2DM [34].

The predominant correlate of AC was distance to work with few people who lived over 1.5 miles from their workplace choosing AC and those living in rural areas less likely to actively commute. Although distance from home to work is not modifiable, AC as the whole or part of the journey could be encouraged by providing more cycleways, by road safety measures to

For peer review only - http://bmjopen.bmj.com/site/about/guidelines.xhtml

limit traffic speed and improving access to parks and green spaces [35][36]. We were unable to examine the association of access to transport as a correlate of active commuting but it can be hypothesised this would correlate strongly with the transport method chosen. Furthermore, we used a crude measure of active commuting (walking or cycling only) and were therefore unable to explore the role of active commuting as a contributor to the overall journey. Individuals who live beyond 3-6 miles from work are unlikely to solely use active commuting modes to travel to work and are more likely to combine active commuting with another form of activity such as the car or public transport. Therefore the role of active commuting as a component of the overall journey should be explored in future research.

In the current study, the results for public transport were equivocal with no apparent observations with physical activity observed. This contrasts with previous research (17) and may be due to an inability to differentiate between the different types of public transport taken and the public transport category also including people who reported mixed modes of motorised travel and active commuting. There are likely to be variations in the energy requirements of different transport modes which should be examined. It could also be hypothesised that walking or activity behaviours associated with public transport are difficult to recall and record in the IPAQ. However, in previous research public transport has been shown to contribute to PA and therefore warrants further investigation.

The final aim of the study was to explore the social and environmental correlates of active commuting behaviour. Those who lived in a rural area were less likely to use active modes of commuting, but no other environmental variables, such as traffic density were shown to be associated. This may be due to an overall lack of variation in the environmental variables and suggests a more diverse sample may be required. For example, there is a lack of inter-individual variation in the measure of air pollution which may explain the lack of association seen.

This study has some major strengths. UK Biobank provides a large, geographically diverse data source allowing multiple social, environmental and employment related factors to be adjusted for in analyses. The study sample is restricted to adults with T2DM and is the first study of its kind to explore these behaviours on such a scale.

The study also has some important limitations. The analyses were cross-sectional and therefore causality cannot be determined. The small numbers of participants reporting AC did not allow walking and cycling to be examined separately. The relative energy demands of these behaviours differ and previous research has suggested these behaviours are performed by different portions of society [37]. The current study was also unable to distinguish between the different modes of public transport. The observed gender difference

For peer review only - http://bmjopen.bmj.com/site/about/guidelines.xhtml

For peer review only

suggests there may be differences in the work and travel patterns of males and females in this study population. The average distance from home to work was almost twice as far for males which may explain why more females reported active modes of travel. The study is further limited by self-report measures of PA and sedentary behaviour.

The study sample is predominantly of White British ethnicity which does not reflect the distribution of type 2 diabetes in the UK limiting the generalisability of the findings. The sample is also limited to those who work and therefore can only examine the role of active commuting on PA and sedentary time. The contribution of non-work active travel may be of more interest for people with T2DM who in general are older than the UK average.

In conclusion, AC is associated with increased PA, and reduced sedentary time in people with type 2 diabetes. This may have a beneficial effect on glucose control and could contribute towards reduced treatment costs. Strategies to increase active commuting in adults with T2DM may increase levels of physical activity, and thus contribute to reducing the burden of this disease.

FUNDING

This project was funded by a small project grant from the National Institute for Health Research Bristol Nutrition Biomedical Research Unit. The funding covered the cost of data access only.

AUTHORS CONTRIBUTIONS

CF, AC and EF made substantial contributions to the conception and design, acquisition of data, or analysis and interpretation of data. All authors drafted the article or revised it critically for important intellectual content. All authors approved the final version of the manuscript to be published.

DATA SHARING STATEMENT

No additional data is available.

COMPETING INTEREST STATEMENT

There are no competing interests to declare by any authors.

REFERENCES

1 Hu FB, Manson JE, Stampfer MJ, *et al.* Diet, lifestyle, and the risk of type 2 diabetes mellitus in women. *N Engl J Med* 2001;**345**:790–7. doi:10.1056/NEJMoa010492

2 Kumari M, Head J, Marmot M, *et al.* Prospective Study of Social and Other Risk Factors for Incidence of Type 2 Diabetes in the Whitehall II Study. *Arch Intern Med*

For peer review only - http://bmjopen.bmj.com/site/about/guidelines.xhtml

2004;**164**:1873. doi:10.1001/archinte.164.17.1873

3 WHO (World Health Organisation). Global Report on Diabetes. *WHO* Published Online First: 2016.http://apps.who.int/iris/bitstream/10665/204871/1/9789241565257_eng.pdf?ua=1 (accessed 22 Jul2016).

4 Hex N, Bartlett C, Wright D, *et al.* Estimating the current and future costs of Type 1 and Type 2 diabetes in the UK, including direct health costs and indirect societal and productivity costs. *Diabet Med* 2012;**29**:855–62. doi:10.1111/j.1464-5491.2012.03698.x

5 Mary G, Caroline H, Naomi H, *et al.* Adult obesity and type 2 diabetes. Published Online First: 2014.www.gov.uk/phe (accessed 6 Jul2016).

6 Sigal RJ, Kenny GP, Wasserman DH, *et al.* Physical activity/exercise and type 2 diabetes: a consensus statement from the American Diabetes Association. *Diabetes Care* 2006;**29**:1433–8. doi:10.2337/dc06-9910

7 Chief Medical Officer. Physical activity guidelines for Adults (19-64 years). 2011.

8 Andrews RC, Cooper AR, Montgomery AA, *et al.* Diet or diet plus physical activity versus usual care in patients with newly diagnosed type 2 diabetes: the Early ACTID randomised controlled trial. *Lancet (London, England)* 2011;**378**:129–39. doi:10.1016/S0140-6736(11)60442-X

9 JAKICIC JM, GREGG E, KNOWLER W, *et al.* Activity Patterns of Obese Adults with Type 2 Diabetes in the Look AHEAD Study. *Med Sci Sport Exerc* 2010;**42**:1995–2005. doi:10.1249/MSS.0b013e3181e054f0

10 Grøntved A, Hu FB. Television viewing and risk of type 2 diabetes, cardiovascular disease, and all-cause mortality: a meta-analysis. *JAMA* 2011;**305**:2448–55. doi:10.1001/jama.2011.812

11 Cassidy S, Chau JY, Catt M, *et al.* Cross-sectional study of diet, physical activity, television viewing and sleep duration in 233,110 adults from the UK Biobank; the behavioural phenotype of cardiovascular disease and type 2 diabetes. *BMJ Open* 2016;**6**:e010038. doi:10.1136/bmjopen-2015-010038

12 Physical activity: walking and cycling | Guidance and guidelines | NICE.

13 FPH. Take action on active travel. Fac. Public Heal. 2008.http://www.fph.org.uk/uploads/Take_action_on_active_travel.pdf (accessed 15 May2016).

14 Laverty research fellow AA, Hérick de Sá student T, Monteiro professor CA. Getting sedentary people moving through active travel. *J Epidemiol Community Heal* 2012;**66**:176–80. doi:10.1136/bmj.h725

15 Goodman A, Hamer M, Chida Y, *et al.* Walking, Cycling and Driving to Work in the English and Welsh 2011 Census: Trends, Socio-Economic Patterning and Relevance to Travel Behaviour in General. *PLoS One* 2013;**8**:e71790. doi:10.1371/journal.pone.0071790

16 Flint E, Cummins S, Sacker A. Associations between active commuting, body fat, and body mass index: population based, cross sectional study in the United Kingdom. BMJ (Clinical Res. ed). 2014. doi:10.1136/bmj.g4887 <http://dx.doi.org/10.1136/bmj.g4887>

17 Flint E, Cummins S. Does active commuting protect against obesity in mid-life?

For peer review only - http://bmjopen.bmj.com/site/about/guidelines.xhtml

Cross-sectional, observational evidence from UK Biobank. *Lancet* Published Online First: 2015. doi:10.1016/S0140-6736(15)00846-6

18 Laverty AA, Mindell JS, Webb EA, *et al.* Active Travel to Work and Cardiovascular Risk Factors in the United Kingdom. *Am J Prev Med* 2013;**45**:282–8. doi:10.1016/j.amepre.2013.04.012

19 Audrey S, Procter S, Cooper AR, *et al.* The contribution of walking to work to adult physical activity levels: a cross sectional study. *Int J Behav Nutr Phys Act* 2014;**11**:37. doi:10.1186/1479-5868-11-37

20 Flint E, Webb E, Cummins S. Change in commute mode and body-mass index: prospective, longitudinal evidence from UK Biobank. *Lancet Public Heal* 2016;**1**:e46–55. doi:10.1016/S2468-2667(16)30006-8

21 Stewart G, Anokye NK, Pokhrel S. What interventions increase commuter cycling? A systematic review. *BMJ Open* 2015;**5**:e007945. doi:10.1136/bmjopen-2015-007945

22 Frank LD, Saelens BE, Powell KE, *et al.* Stepping towards causation: Do built environments or neighborhood and travel preferences explain physical activity, driving, and obesity? *Soc Sci Med* 2007;**65**:1898–914. doi:10.1016/j.socscimed.2007.05.053

23 Allen NE, Sudlow C, Peakman T, *et al.* UK biobank data: come and get it. *Sci Transl Med* 2014;**6**:224ed4. doi:10.1126/scitranslmed.3008601

24 Biobank U. Rationale, design and development of a large-scale prospective resource. UK Biobank. 2007.http://www.ukbiobank.ac.uk/resources/

25 Craig CL, Marshall AL, Sjöström M, *et al.* International physical activity questionnaire: 12-country reliability and validity. *Med Sci Sports Exerc* 2003;**35**:1381–95. doi:10.1249/01.MSS.0000078924.61453.FB

26 IPAQ. Guidelines for Data Processing and Analysis of the International Physical Activity Questionnaire (IPAQ) - Short Form,.

27 Small area health statistics unit. www.sashu.org

28 BioSHaRE EU Determinants of Health Project. http://bioshare.eu

29 Eeftens M, Beelen R, Hoogh K de, *et al.* Development of Land Use Regression Models for PM2.5, PM2.5 Absorbance, PM10 and PMcoarse in 20 European Study Areas; Results of the ESCAPE Project. 2012.

30 StataCorp 2015. Stata statistical software: Release 14.

31 Brocklebank LA, Falconer CL, Page AS, *et al.* Accelerometer-measured sedentary time and cardiometabolic biomarkers: A systematic review. *Prev Med (Baltim)* Published Online First: 2015. doi:10.1016/j.ypmed.2015.04.013

32 Owen N, Sparling PB, Healy GN, *et al.* Sedentary behavior: emerging evidence for a new health risk. *Mayo Clin Proc* 2010;**85**:1138–41. doi:10.4065/mcp.2010.0444

33 Falconer CL, Cooper AR, Walhin JP, *et al.* Sedentary time and markers of inflammation in people with newly diagnosed type 2 diabetes. *Nutr Metab Cardiovasc Dis* 2014;**24**:956–62. doi:10.1016/j.numecd.2014.03.009

34 Falconer CL, Page AS, Andrews RC, *et al.* The Potential Impact of Displacing Sedentary Time in Adults with Type 2 Diabetes. *Med Sci Sports Exerc* 2015;**47**:2070–5. doi:10.1249/MSS.0000000000000651

For peer review only - http://bmjopen.bmj.com/site/about/guidelines.xhtml

35 Mackenbach JD, Rutter H, Compernolle S, *et al.* Obesogenic environments: a systematic review of the association between the physical environment and adult weight status, the SPOTLIGHT project. *BMC Public Health* 2014;**14**:233. doi:10.1186/1471-2458-14-233

36 Tomlinson P, Hewitt S, Blackshaw N. Joining up health and planning: how Joint Strategic Needs Assessment (JSNA) can inform health and wellbeing strategies and spatial planning. *Perspect Public Health* 2013;**133**:254–62. doi:10.1177/1757913913488331

37 Mytton OT, Panter J, Ogilvie D. Longitudinal associations of active commuting with wellbeing and sickness absence. *Prev Med (Baltim)* 2016;**84**:19–26. doi:10.1016/j.ypmed.2015.12.010

For peer review only

For peer review only - http://bmjopen.bmj.com/site/about/guidelines.xhtml

For peer review only

For peer review only - http://bmjopen.bmj.com/site/about/guidelines.xhtml

For peer review only

For peer review only - http://bmjopen.bmj.com/site/about/guidelines.xhtml

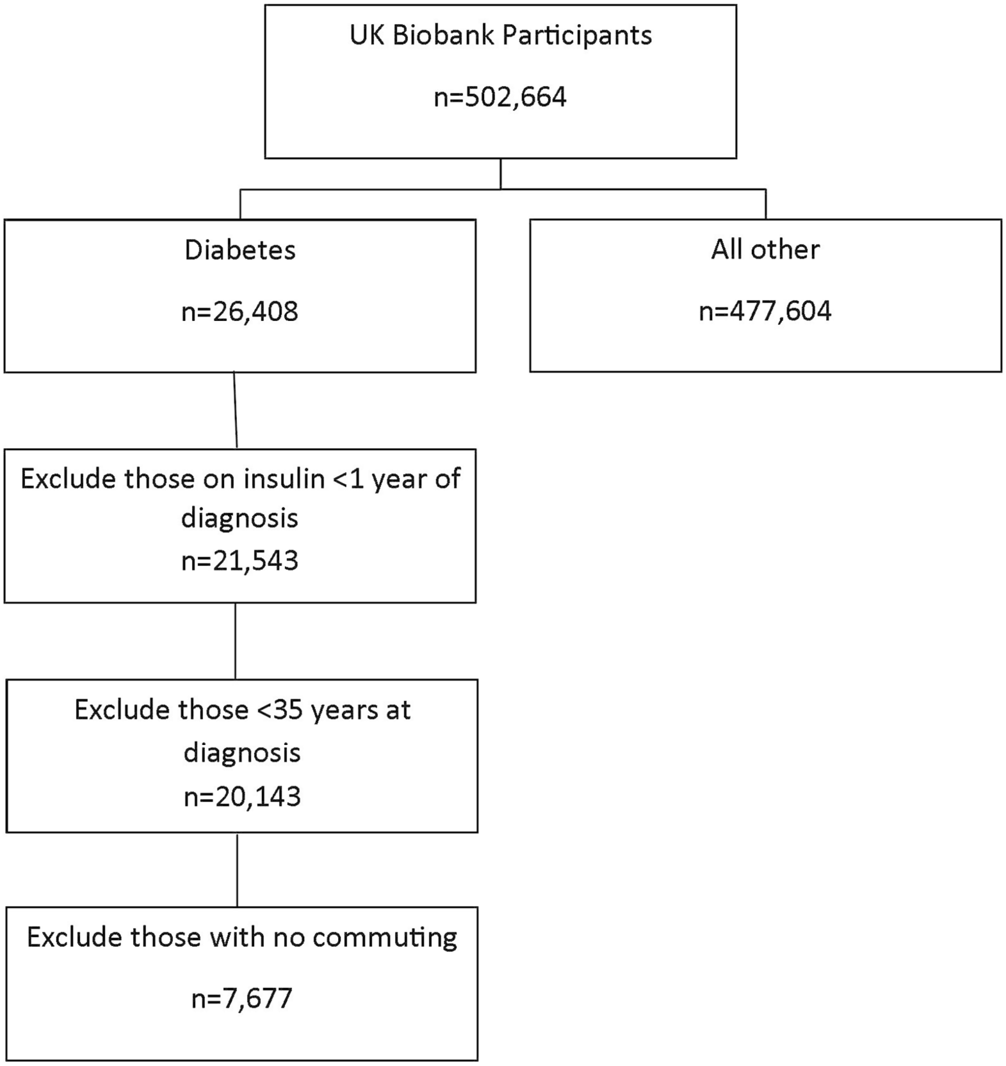

Figure 1. Flow chart demonstrating how population sample was identified.

135x142mm (300 x 300 DPI)

STROBE Statement—checklist of items that should be included in reports of observational studies

	Item No	Recommendation	Page number
**Title and abstract**	1	(*a*) Indicate the study's design with a commonly used term in the title or the abstract	1
		(*b*) Provide in the abstract an informative and balanced summary of what was done and what was found	2
**Introduction**			
Background/rationale	2	Explain the scientific background and rationale for the investigation being reported	4-5
Objectives	3	State specific objectives, including any prespecified hypotheses	5
**Methods**			
Study design	4	Present key elements of study design early in the paper	6
Setting	5	Describe the setting, locations, and relevant dates, including periods of recruitment, exposure, follow-up, and data collection	6
Participants	6	(*a*) *Cohort study*—Give the eligibility criteria, and the sources and methods of selection of participants. Describe methods of follow-up<br>*Case-control study*—Give the eligibility criteria, and the sources and methods of case ascertainment and control selection. Give the rationale for the choice of cases and controls<br>*Cross-sectional study*—Give the eligibility criteria, and the sources and methods of selection of participants	6
		(*b*) *Cohort study*—For matched studies, give matching criteria and number of exposed and unexposed<br>*Case-control study*—For matched studies, give matching criteria and the number of controls per case	
Variables	7	Clearly define all outcomes, exposures, predictors, potential confounders, and effect modifiers. Give diagnostic criteria, if applicable	6-7
Data sources/ measurement	8*	For each variable of interest, give sources of data and details of methods of assessment (measurement). Describe comparability of assessment methods if there is more than one group	6-7
Bias	9	Describe any efforts to address potential sources of bias	7
Study size	10	Explain how the study size was arrived at	NA
Quantitative variables	11	Explain how quantitative variables were handled in the analyses. If applicable, describe which groupings were chosen and why	8
Statistical methods	12	(*a*) Describe all statistical methods, including those used to control for confounding	8
		(*b*) Describe any methods used to examine subgroups and interactions	88
		(*c*) Explain how missing data were addressed	
		(*d*) *Cohort study*—If applicable, explain how loss to follow-up was addressed<br>*Case-control study*—If applicable, explain how matching of cases and controls was addressed<br>*Cross-sectional study*—If applicable, describe analytical methods taking account of sampling strategy	8
		(*e*) Describe any sensitivity analyses	8

Continued on next page

For peer review only - http://bmjopen.bmj.com/site/about/guidelines.xhtml

**Results**			
Participants	13*	(a) Report numbers of individuals at each stage of study—eg numbers potentially eligible, examined for eligibility, confirmed eligible, included in the study, completing follow-up, and analysed	
		(b) Give reasons for non-participation at each stage	
		(c) Consider use of a flow diagram	
Descriptive data	14*	(a) Give characteristics of study participants (eg demographic, clinical, social) and information on exposures and potential confounders	8
		(b) Indicate number of participants with missing data for each variable of interest	
		(c) *Cohort study*—Summarise follow-up time (eg, average and total amount)	
Outcome data	15*	*Cohort study*—Report numbers of outcome events or summary measures over time	8
		Case-control study—Report numbers in each exposure category, or summary measures of exposure	
		Cross-sectional study—Report numbers of outcome events or summary measures	
Main results	16	(*a*) Give unadjusted estimates and, if applicable, confounder-adjusted estimates and their precision (eg, 95% confidence interval). Make clear which confounders were adjusted for and why they were included	8-10
		(*b*) Report category boundaries when continuous variables were categorized	
		(*c*) If relevant, consider translating estimates of relative risk into absolute risk for a meaningful time period	
Other analyses	17	Report other analyses done—eg analyses of subgroups and interactions, and sensitivity analyses	8-10
**Discussion**			
Key results	18	Summarise key results with reference to study objectives	10-11
Limitations	19	Discuss limitations of the study, taking into account sources of potential bias or imprecision. Discuss both direction and magnitude of any potential bias	12
Interpretation	20	Give a cautious overall interpretation of results considering objectives, limitations, multiplicity of analyses, results from similar studies, and other relevant evidence	12
Generalisability	21	Discuss the generalisability (external validity) of the study results	12
**Other information**			
Funding	22	Give the source of funding and the role of the funders for the present study and, if applicable, for the original study on which the present article is based	13

*Give information separately for cases and controls in case-control studies and, if applicable, for exposed and unexposed groups in cohort and cross-sectional studies.

Note: An Explanation and Elaboration article discusses each checklist item and gives methodological background and published examples of transparent reporting. The STROBE checklist is best used in conjunction with this article (freely available on the Web sites of PLoS Medicine at http://www.plosmedicine.org/, Annals of Internal Medicine at http://www.annals.org/, and Epidemiology at http://www.epidem.com/). Information on the STROBE Initiative is available at www.strobe-statement.org.

For peer review only

For peer review only - http://bmjopen.bmj.com/site/about/guidelines.xhtml
